# Supplementary material for: Duration of External Neck Stabilisation (DENS) following odontoid fracture in older or frail adults: protocol for a randomised controlled trial of collar versus no collar
Source: BMJ Open. 2022 Jul 14;12(7):e057753. doi: 10.1136/bmjopen-2021-057753 (PMC9295672; doi:10.1136/bmjopen-2021-057753)
Supplement: Author's manuscript [file bmjopen-2021-057753.draft_revisions.pdf]

BMJ Open

BMJ Open is committed to open peer review. As part of this commitment we make the peer review history of every article we publish publicly available.

When an article is published we post the peer reviewers' comments and the authors' responses online. We also post the versions of the paper that were used during peer review. These are the versions that the peer review comments apply to.

The versions of the paper that follow are the versions that were submitted during the peer review process. They are not the versions of record or the final published versions. They should not be cited or distributed as the published version of this manuscript.

BMJ Open is an open access journal and the full, final, typeset and author-corrected version of record of the manuscript is available on our site with no access controls, subscription charges or pay-per-view fees (http://bmjopen.bmj.com).

If you have any questions on BMJ Open's open peer review process please email info.bmjopen@bmj.com

BMJ Open

Duration of External Neck Stabilisation (DENS) following odontoid fracture in older or frail adults: protocol for a randomised controlled trial of collar versus no collar

Journal:	*BMJ Open*
Manuscript ID	bmjopen-2021-057753
Article Type:	Protocol
Date Submitted by the Author:	25-Sep-2021
Complete List of Authors:	Woodfield, Julie; NHS Lothian, Department of Clinical Neurosciences; The University of Edinburgh Centre for Clinical Brain Sciences, Translational Neurosurgery<br>Edlmann, Ellie; Derriford Hospital, Southwest Neurosurgical Centre; University of Plymouth, Peninsula Medical School<br>Black, Polly; NHS Lothian, Emergency Medicine Research Group (EMERGE)<br>Boyd, Julia; Edinburgh Clinical Trials Unit<br>Copley, Phillip; NHS Lothian, Department of Clinical Neurosciences<br>Cranswick, Gina; University of Edinburgh , Edinburgh Clinical Trials Unit<br>Eborall, Helen; University of Edinburgh , Usher Institute<br>Keerie, Catriona; University of Edinburgh College of Medicine and Veterinary Medicine, Edinburgh Clinical Trials Unit<br>Khan, Sadaquate; NHS Lothian, Department of Clinical Neurosciences<br>Lawton, Julia; University of Edinburgh, Centre for Population Health Sciences<br>Lowe, David; Queen Elizabeth University Hospital Campus, Emergency Departmebnt; University of Glasgow Institute of Health and Wellbeing<br>Norrie, John; Edinburgh Clinical Trials Unit, University of Edinburgh No. 9, Bioquarter<br>Niven, Angela; Edinburgh Clinical Trials Unit<br>Reed, Matthew; Royal Infirmary of Edinburgh, Emergency Medicine Research Group; The University of Edinburgh Usher Institute of Population Health Sciences and Informatics<br>Shenkin, Susan; University of Edinburgh College of Medicine and Veterinary Medicine, Advanced Care Research Centre; The University of Edinburgh Usher Institute of Population Health Sciences and Informatics<br>Statham, Patrick; NHS Lothian, Department of Clinical Neurosciences<br>Stoddart, Andrew; The University Of Edinburgh, Edinburgh Health Services Research Unit<br>Tomlinson, James; Sheffield Teaching Hospitals NHS Foundation Trust<br>Brennan, Paul; The University of Edinburgh Centre for Clinical Brain Sciences, Translational Neurosurgery; NHS Lothian, Department of Clinical Neurosciences
Keywords:	Protocols & guidelines < HEALTH SERVICES ADMINISTRATION & MANAGEMENT, NEUROSURGERY, ORTHOPAEDIC & TRAUMA SURGERY

SCHOLARONE™
Manuscripts

Title

Duration of External Neck Stabilisation (DENS) following odontoid fracture in older or frail adults: protocol for a randomised controlled trial of collar versus no collar

Authors

Julie Woodfield,[1,2] Ellie Edlmann,[3,4] Polly Black,[5] Julia Boyd,[6] Phillip Correia Copley,[2] Gina Cranswick,[6] Helen Eborall,[7] Catriona Keerie,[6] Sadaquate Khan,[2] Julia Lawton,[7] David J Lowe,[8,9] John Norrie,[6] Angela Niven,[6] Matthew J Reed,[5,7] Susan D Shenkin,[7,10] Patrick FX Statham,[2] Andrew Stoddart,[6] James Tomlinson,[11] Paul M Brennan[1,2]

1. Translational Neurosurgery, Centre for Clinical Brain Sciences, University of Edinburgh, Edinburgh, UK
2. Department of Clinical Neurosciences, NHS Lothian, Edinburgh, UK
3. Southwest Neurosurgical Centre, Derriford Hospital, Plymouth, UK
4. Peninsula Medical School, University of Plymouth, Plymouth, UK
5. Emergency Medicine Research Group Edinburgh (EMERGE), Department of Emergency Medicine, Royal Infirmary of Edinburgh, Edinburgh, UK
6. Edinburgh Clinical Trials Unit, Edinburgh, UK
7. Usher Institute, University of Edinburgh, Edinburgh, UK
8. Emergency Department, Queen Elizabeth University Hospital, NHS Greater Glasgow and Clyde, Glasgow, UK
9. Institute of Health and Wellbeing, University of Glasgow, Glasgow, UK
10. Advanced Care Research Centre, University of Edinburgh, Edinburgh
11. Sheffield Teaching Hospitals NHS Foundation Trust, Sheffield, UK

Corresponding Author

Mr Paul Brennan, Translational Neurosurgery, Centre for Clinical Brain Sciences, Chancellor's Building, 49 Little France Crescent, Edinburgh, EH16 4SB, United Kingdom
Paul.brennan@ed.ac.uk
Tel: +44 (0) 131 537 1000

Word Count: 3415

Abstract Word Count: 300

For peer review only - http://bmjopen.bmj.com/site/about/guidelines.xhtml

ABSTRACT

Introduction

Fractures of the odontoid process frequently result from low impact falls in frail or older adults. These are increasing in incidence and importance as the population ages. In the UK, odontoid fractures in older adults are usually managed in hard collars to immobilise the fracture and promote bony healing. However, bony healing does not always occur in older adults, and bony healing is not associated with quality of life, functional, or pain outcomes. Further, hard collars can cause complications such as skin pressure ulcers, swallowing difficulties, and difficulties with personal care. We hypothesise that management with no immobilisation may be superior to management in a hard collar for older or frail adults with odontoid fractures.

Methods & Analyses

This is the protocol for the Duration of External Neck Stabilisation (DENS) trial - a non-blinded randomised controlled trial comparing management in a hard collar with management without a collar for older (≥65 years) or frail (Rockwood clinical frailty scale ≥5) adults with a new odontoid fracture. 887 neurologically intact participants with any odontoid process fracture type will be randomised to continuing with a hard collar (standard care) or removal of the collar (intervention). The primary outcome is quality of life measured using the EQ-5D-5L at 12 weeks. Secondary outcomes include pain scores, neck disability index, health and social care use and costs, and mortality.

Ethics & Dissemination

Informed consent for participation will be sought from those able to provide it. We will also include those who lack capacity to ensure representativeness of frail and acutely unwell older adults. Results will be disseminated via scientific publication, lay summary, and visual abstract.

The DENS trial received a favourable ethical opinion from the Scotland A Research Ethics Committee (21/SS/0036) and the Leeds West Research Ethics Committee (21/YH/0141).

Registration Details

Registered at ClinicalTrials.gov (NCT04895644).

For peer review only

For peer review only - http://bmjopen.bmj.com/site/about/guidelines.xhtml

Strengths & Limitations of this study

- The management of odontoid process fractures is of increasing importance in an aging population at risk of low impact falls.
- The DENS trial is a non-blinded randomised controlled trial to compare the use of hard collars with no immobilisation.
- A patient relevant quality of life primary outcome measure (EQ-5D-5L) has been chosen.
- Health economic analysis and embedded qualitative study methods assess the wider implications of collar use in the older or frail population.
- The trial employs inclusive recruitment of all fracture types in older or frail patients including those who lack capacity.

Key Words
Odontoid process, odontoid peg, C2, cervical fracture, axis, dens, older adults, elderly, frailty, low-impact falls, cervical immobilisation, hard collar

For peer review only

For peer review only - http://bmjopen.bmj.com/site/about/guidelines.xhtml

INTRODUCTION

Background and Rationale

Odontoid process (dens) fractures occur following low impact falls in frail and older people, and are increasing in incidence as the population ages.[1-3] The Trauma Audit and Research Network (TARN) database identified at least 1,700 odontoid fractures each year in England and Wales, 85% in people over 65 years. One-year mortality following an odontoid fracture is 20-50%, similar to or higher than following a hip fracture, likely reflecting the underlying frailty and health status of those at risk of low impact falls.[4-7]

In the UK, 85-90% of frail or older people with a new odontoid fracture are managed non-operatively, most with 6-12 weeks immobilisation in a hard collar.[8-10] This aims to promote bony fusion and prevent neurological deterioration or instability pain.[1 5 11] However, hard collars only restrict 40-50% of neck movements,[12 13] and bony fusion rates of 20-80% are variable across fracture types, management, age, and frailty.[1 5 11 14-18] Further, bony fusion may not be associated with pain, quality of life, mortality, or functional outcomes in older people,[8 19 20] and late neurological deterioration is very rare.[5 11 21] Hard collars can cause skin pressure ulcers and difficulties with swallowing, breathing, and personal care, all of which can affect quality of life (QoL).[10 22] Additional health and social care input is often required to assist patients with activities of daily living. If fibrous union, or lack of bony fusion is an acceptable outcome and hard collars can negatively impact QoL, then management of an odontoid fracture with a hard collar may be causing unnecessary harm and negatively impacting on QoL in a group for whom the short to medium term QoL may be more important than long term outcomes.

Spinal surgeons responding to a UK survey variably reported managing odontoid fractures without any immobilisation or removing hard collars in frail or older patients who were unable to tolerate a collar, had a short life expectancy, or suffered complications.[10] Ninety percent supported randomisation to management with or without a collar to determine optimum treatment.[10] Further, feedback from patients managed in collars supports exploring whether collars are necessary for odontoid process fractures.

The Duration of External Neck Stabilisation (DENS) trial will test the hypothesis that management of odontoid process fractures without immobilisation is associated with improved QoL compared to management in a hard collar for 12 weeks.

Aims and Objectives

The primary aim is to determine the difference in QoL measured using the EQ-5D-5L at 12 weeks between frail or older patients with odontoid process fractures managed with a hard collar (standard care) or without any immobilisation (intervention).

Secondary aims include assessing differences in neck pain, functional recovery, complications, radiological findings, mortality, and overall health-care use and costs, up to one year post injury.

A nested qualitative study will explore trial recruitment experiences from the perspectives of patients, carers, and healthcare professionals, and explore adherence to group allocation.

For peer review only - http://bmjopen.bmj.com/site/about/guidelines.xhtml

Findings from the qualitative study will inform recruitment and consent procedures and aid interpretation of trial results.

METHODS AND ANALYSIS

Study design

The DENS trial is a non-blinded randomised controlled trial with a nested qualitative study comparing early hard collar removal (intervention) with treatment in a hard collar for 12 weeks (standard care) in older or frail adults with a new odontoid process fracture. Figure 1 shows the trial flow chart.

Study Eligibility

Inclusion Criteria:

- Aged ≥65 years, or Rockwood clinical frailty scale (CFS) of ≥5 (at least mildly frail: help needed in high order instrumental activities of daily living)[23]
- Recent odontoid process fracture of any type[24] secondary to low impact trauma (any degree of fracture angulation, displacement or canal narrowing)
- Recruited within 3 weeks of injury
- Determined by consultant spinal surgeon as suitable for standard treatment in hard collar or treatment without a collar

Exclusion Criteria:

- Fracture sustained in a high-impact injury (e.g. fall from more than 6m, motor vehicle accident)
- New neurological deficit attributable to the fracture
- Unable to tolerate a hard collar
- Additional (non-odontoid process) cervical spine fracture requiring hard collar or surgical treatment
- Underlying condition with risk of spinal instability (e.g. ankylosing spondylitis)
- Fracture sustained more than three weeks prior to recruitment
- Not expected to survive to hospital discharge

A consultant radiologist report of an odontoid process fracture on CT and confirmation of eligibility by a consultant spinal surgeon (orthopaedic or neurosurgical) is required. Patients with additional cervical spine fractures are eligible provided the consultant spinal surgeon determines the fracture(s) are suitable for management without a hard collar. There are no exclusions for other injuries (e.g. fractured femur). Co-enrolment in other studies is permitted where this does not affect the DENS trial randomisation allocation or outcome measure assessment, and where this is not expected to burden the participant.

Participant Recruitment

Potential participants will be identified by clinicians following presentation to Emergency Departments (EDs) or acute assessment units, through review of acute cervical spine CT reports, acute hospital attendance, or through referrals or admissions to spinal services in the UK. An anonymised screening log will be kept.

For peer review only - http://bmjopen.bmj.com/site/about/guidelines.xhtml
For peer review only

Any neck stabilisation can be applied as part of standard care prior to randomisation, including; trauma collar, blocks, spinal board, padded hard collar (e.g. Miami J, Aspen, Philadelphia) or soft collar. Standard care treatment, such as removal from spinal boards and placement of a padded hard collar will occur as usual, and will not be delayed by trial assessment and participation.

Eligibility assessment and randomisation will take place as soon as possible after injury (target within 48 hours) to maximise the impact of study intervention. However, falls in older and frail people are usually multifactorial,[25 26] and conditions leading to the fall or resulting from the fall may affect capacity and fitness to participate. Recruitment can occur up to 3 weeks after the injury to provide flexibility for those presenting late, facilitate participation in those who are acutely unwell, and ensure time for involvement of patient representatives where needed.

The nested qualitative study will include healthcare professionals involved in patient recruitment, study participants, carers of participants lacking capacity, and those who decline trial participation.

Randomisation and Interventions
Web-based randomisation will occur with a minimisation algorithm based on:

- Age at randomisation (<75 versus ≥75 years)
- Odontoid Fracture type (I-III)[24]
- Frailty (CFS ≥5 versus <5)[23]

The first participant will be allocated to one arm with a probability of 0.5 and subsequent participants will be allocated with a probability of 0.8 to the group which minimises differences of the above variables between the two trial arms. Treatment allocation will be disclosed to the local research team via the web interface following randomisation.

The standard care arm will involve 12 weeks of hard collar treatment, following local usual choice of hard collar and local usual arrangements for collar care. For those allocated to the intervention (collar removal) arm, the collar will be removed as soon as possible, but may be weaned over several days if required for optimising analgesia. Removal of a collar prior to 12 weeks in the hard collar arm or wearing of a collar in the no collar arm will be recorded with explanation. Soft collars will not be used in either arm.

Outcome Measures
The primary outcome measure is the EQ-5D-5L at 12 weeks post-randomisation.
Secondary outcome measures are:

- 2 & 6 weeks: EQ-5D-5L, neck disability index (NDI), neck pain visual analogue score (VAS), mobility, adverse events (AEs)
- 12 weeks: NDI, neck pain VAS, AEs, compliance with allocation, analgesia use, length of hospital stay, discharge destination, bony fusion / stability on imaging
- 6 & 12 months: EQ-5D-5L, NDI, neck pain VAS, complications, mortality, health service use and costs

Trial assessments

For peer review only - http://bmjopen.bmj.com/site/about/guidelines.xhtml

Assessments and timing of data collection are shown in Table 1. Baseline data on demographics, comorbidities, injury, radiological findings and inpatient management will be recorded. Patient diaries will be used to collect pain scores, analgesia and collar use. Standard local clinical follow up will occur. Research assessments will be carried out at 2, 6 and 12 weeks by the local research team and by a blinded member of the central research team at 6 and 12 months. Assessments will occur via telephone interviews, postal or online questionnaires, or during standard care appointments. A proxy can provide information on a patient's behalf when required. Up to three reminder telephone calls will be made to maximise completion.

Table 1: **Timing of Study Assessments.**
Assessments undertaken as standard care are shown with x. Study specific assessments are shown with *. Assessments may occur within the following time windows: 2 weeks (±1 week), 6 and 12 weeks (± 2 weeks) and 6 and 12 months (± 4 weeks). Fracture type refers to that of Anderson & D'Alonzo.[24] (CFS: Rockwood clinical frailty scale;[23] VAS: visual analogue score; NDI: neck disability index; AEs: adverse events)

	**Timing from Randomisation**						
	Baseline	Discharge	2 weeks	6 weeks	12 weeks	6 months	12 months
**Assessment**							
Eligibility Assessment	*						
Consent	*						
**Demographics**							
Baseline data	*						
CFS	*						
**Imaging**							
Fracture Type	x						
Imaging Type	x				x		
**Outcomes**							
EQ-5D-5L			*	*	*	*	*
EQ-VAS			*	*	*	*	*
Neck pain VAS			*	*	*	*	*
NDI			*	*	*	*	*
AEs		*			*		
Injury-related complications						*	*
Collar use		*	*	*	*		
Grip strength/bioimpedance	*				*		
Service use			*	*	*	*	*
Analgesia use			*	*	*		
Mortality					*	*	*

Where available, bioimpedence and grip strength will be measured objectively to support interpretation of the CFS. Approximately 25 patients in the collar arm will use temperature sensors to assess compliance with the collar (Thermochron iButtons).[27] Long term follow up of mortality and hospital admissions using anonymised data without participant contact will occur for 5 years.

For peer review only - http://bmjopen.bmj.com/site/about/guidelines.xhtml

Qualitative researchers will interview healthcare professionals about their recruitment experiences, perceptions of why patients decline or consent to participation, and reasons for not approaching eligible patients. Patients and caregivers will be interviewed at two time points – following randomisation and 12 weeks later. Post-randomisation interviews will explore reasons for taking part or declining participation, views about trial recruitment approaches, hopes and expectations regarding trial participation, and any anticipated difficulties or concerns adhering to treatment allocation. Follow-up interviews will explore treatment adherence, benefits or burdens of the allocated treatment, and perceptions of the impact on QoL and recovery.

Sample Size

The recruitment target is 887 participants from around 25 UK sites over 2 years. An unadjusted sample size was calculated based on 90% power and a 5% significance level, using a two-sided two-sample t-test, for an effect size of 1/6 (0.05 minimum clinically important difference / 0.3 standard deviation) on the EQ-5D-5L at 12 weeks. This results in a sample size of 1514, increasing to 1893 assuming 20% missing data. This sample size can be reduced by accounting for correlation between baseline covariates (age, CFS) and 12 week EQ-5D-5L. It is anticipated that this correlation will be around 0.5, allowing a 25% reduction in sample size. Higher correlation will allow a larger reduction.[28] Further, the post-randomisation measures of EQ-5D-5L at 2, 6 and 12 weeks, are assumed to have a serial correlation of 0.44 which allows a further 37.5% reduction in sample size.[28 29] Including these reductions leads to the final target sample size of 887, with 1:1 allocation.

To check these assumptions, a sample size re-estimation will be modelled using a simulation approach based on 12 week outcome data from the first 300 participants.[28] Due to the conservative assumptions made, particularly the assumed standard deviation, the probability of increasing the sample size is small, and there is a larger probability it can be reduced.

Based on audits in two UK neurosurgical units, and assuming a 50% recruitment rate with staggered site opening, the target recruitment rate is 1.6 patients per site per month. An internal pilot will assess recruitment feasibility, targeting 132 patients (15% total) across 18 centres in 9 months. Progression criteria for the pilot are shown in Table 2.

Table 2: Study Progression Criteria

Study progression criteria for the internal pilot to assess recruitment feasibility after the first 9 months of recruitment. The traffic light categories will lead to the following actions. Green: continue unchanged. Amber: make changes, including adding more sites or increasing the time period for recruitment. Red: continue stopping as study may not feasible, unless identifiable and rectifiable causes are identified. Adherence failure includes study withdrawals and cross-over (removing a collar in the collar arm, or wearing a collar in the no collar arm).

For peer review only - http://bmjopen.bmj.com/site/about/guidelines.xhtml

	Category		
	Red	Amber	Green
Total number recruited	≤108	109-131	≥132
Recruitment threshold (%)	≤82%	83%-99%	100%
Recruitment rate (patients per site per month)	1.07	1.33	1.63
Number of sites open	<5	5-10	>10
Adherence failure	>20%	11-20%	≤10%

The qualitative study will recruit approximately 20 healthcare professionals and 30 patients or carers from a cross section of sites. Purposive sampling will be used to ensure representation of different ages, socio-economic backgrounds, cognitions, and residential setting amongst patients. Sampling decisions may be revised in light of emerging findings.

Data Analysis Plan

The primary data analysis will be by intention to treat. A 5% two-sided significance level will be used throughout. A detailed statistical analysis plan will be developed by the study statisticians and finalised prior to locking of the trial database.

Analysis of the primary outcome will be a repeated measures analysis of covariance, including terms for treatment arm and the EQ-5D-5L responses at 2, 6 and 12 weeks. Adjustments will be made for the variables included in the randomisation minimisation algorithm. Adjustment for study site will be included as a random effect, if appropriate. The repeated measures approach enables estimation of an intervention effect at week 12 post-randomisation (primary outcome), and an overall assessment of the effect of the intervention over the 12-week period.

The 12-week NDI and VAS will be analysed in a similar manner to the EQ-5D-5L. It is anticipated that results will underpin any between group differences seen in EQ-5D-5L, confirming and strengthening the clinical interpretation of the findings. All other secondary outcomes will be analysed with statistical models appropriate to the distribution of the outcome. Where there are data recorded on multiple occasions post randomisation we may use an appropriate repeated measures model. Exploratory subgroup analyses by fracture type (I, II and III),[24] CFS (<5 versus ≥5)[23] and age (<75 versus ≥ 75 years) will be performed. The influence of any missing data on the robustness of the findings will be examined e.g. using multiple imputation models under a missing at random assumption. Screening logs will be analysed to assess generalisability of results.

Interview data will be analysed thematically using the method of constant comparison. To ensure rigour, several individuals will be involved in data analysis and coding. An interactive working group will be set up, comprising the qualitative research team, co-investigators, the trial manager, PPI representatives, and at least one healthcare professional from each of the sites involved in the pilot phase. A ‘what, so what, now what’ approach[30] will be used to

For peer review only - http://bmjopen.bmj.com/site/about/guidelines.xhtml

translate qualitative findings and other experiences shared by group members into tangible recommendations.

A 12 month within trial health economic analysis will be undertaken based on National Institute for Health and Care Excellence (NICE) reference case recommendations to maximise UK policy relevance.[31] This will include: adoption of a National Health Service (NHS) and Personal Social Services decision perspective; cost-utility approach for primary analysis (results presented in terms of incremental cost per quality adjusted life year (QALY), derived from EQ-5D-5L data with an area under the curve approach, omitting baseline); discount rate of 3.5% for both costs and QALYs (where applicable); and use of probabilistic sensitivity analysis, to generate cost effectiveness acceptability curves via the recycled predictions technique.[31 32] Choice of primary analysis cost per QALY threshold and EQ-5D-5L scoring algorithm will match NICE preferences at the time of data lock. Resource use will be combined with standard UK price weights to generate costs.[33 34] The latest financial year for which at least one study participant provides data and prices are available will be selected as base year. Univariate mean QALYs, resource use, and costs will be presented for each trial arm alongside differences in means and associated 95% confidence intervals. Multivariate analysis of both QALYs and total costs, will also be presented controlling for baseline costs where available, and minimisation variables. Missing data will be imputed using appropriate techniques depending on degree of missingness, likely multiple imputation by chained equations.

Patient and Public Involvement

Patients and the public have played an active role in developing the grant application and project protocol, are co-applicants on the grant and are represented on the Trial Steering Committee. We asked older people with odontoid fractures and members of a PPI group for their opinion on EQ-5D-5L and NDI as possible primary outcome measures. Older patients with odontoid fractures managed to complete the EQ-5D-5L, whilst some struggled with the NDI, and described some of the NDI domains as irrelevant. PPI input identified frailty rather than age as more influential of daily function, leading to inclusion criteria including both. A lay group also reviewed and rewrote the lay summary of the trial and had input into its likely acceptability. At the end of the study, we will produce a plain language summary of the study results and a visual abstract, in conjunction with participants and our PPI partners.

ETHICS AND DISSEMINATION

The DENS trial received a favourable ethical opinion from the Scotland A Research Ethics Committee (REC) on the 10th June 2021 (21/SS/0036) and the Leeds West REC (on behalf of England and Wales) on the 29th July 2021 (21/YH/0141).

Participant Capacity and Consent

The study member obtaining consent will explain and clarify all relevant information, either face-to-face, or via telephone or video call, and give patients the time they need to decide whether to participate. Informed consent will be signed and dated by participants or their representative, and a study team member. Verbal consent from a participant or their

For peer review only - http://bmjopen.bmj.com/site/about/guidelines.xhtml

representative, signed and witnessed by a study team member will also be valid. Participants may withdraw consent at any time without reason, without affecting their care.

Patients diagnosed with odontoid fractures may lack capacity. Recruitment of these patients is necessary to aid generalisability of the trial outcome and to allow equal access to any benefits of participation. All consent procedures will adhere to the Adults with Incapacity (Scotland) Act 2000, the 2005 Mental Capacity Act, the Mental Capacity Act (Northern Ireland) 2016, and their codes of practice. For patients in Scotland who lack capacity, the patient's nearest relative, welfare attorney or guardian can provide consent. For patients in England, Wales and Northern Ireland who lack capacity, a personal consultee (relative or close friend) can provide consent. Where possible this will be written and signed, otherwise clear documentation of the discussion and named persons involved will be accepted.

If a participant without capacity regains capacity, informed consent will be sought to continue in the trial. In Scotland, if a participant has given informed consent, but loses capacity, they will remain in the study. In England, Wales, and Northern Ireland, if a participant has already given informed consent, but loses capacity, they will remain in the study only if consent is then given by a consultee.

Safety Considerations

Adverse Events (AEs) will be recorded up to 12-weeks. Expected AEs relating to hard collar use or odontoid process fractures include skin pressure ulcers, dysphagia, and recurrent falls. All AEs (expected or not) will be reviewed by the local principal investigator and reported to the data monitoring committee (DMC). Serious Adverse Events (SAEs) will also be reported to the trial sponsor, who will notify the REC. Outcome data, AEs and SAEs, will be routinely reviewed by the DMC. The DMC is independent and oversees trial safety and data monitoring. If the DMC assesses that the risk-benefit balance is significantly changed by any new safety information, amendments to the trial will be made.

The trial funder (NIHR) and co-sponsors (University of Edinburgh and NHS Lothian) did not influence trial design. The sponsor ensures that data collection, management and trial monitoring are conducted appropriately and has overall responsibility for the study. Neither the funder nor the sponsor will have ultimate authority over writing of the report or the decision to submit the report for publication.

The trial will be coordinated by the central trial team based at the Edinburgh Clinical Trials Unit (ECTU), overseen by a Trial Management Group consisting of key trial members. Regular reports will be made to the Trial Steering Committee.

The Protocol has been designed by the Chief Investigator and collaborators. The University of Edinburgh has insurance for negligent harm caused by poor protocol design, and participating sites have their own insurance and NHS indemnity for clinical negligence and other negligent harm.

Data Management

Participant records will be identified using a unique study identification number. All records will be kept in a secure storage area with limited access. Data will be entered anonymously

For peer review only - http://bmjopen.bmj.com/site/about/guidelines.xhtml

into a secure database created and maintained by ECTU, stored on a secure server at The University of Edinburgh compliant with all relevant regulations. Trained and delegated members of the research team will have password protected logins for their own centre. Data generated from the iButton sensors is anonymised using a numerical code, which will be recorded on the database. Interviews for the qualitative study will be digitally recorded using a device encrypted to AES256 standard. Imaging will be stored and transferred within the NHS network. Only anonymised scans will be processed outside the NHS network. All investigators and study site staff will comply with the requirements of the appropriate data protection legislation (including the General Data Protection Regulation and Data Protection Act 2018) regarding collection, storage, processing and disclosure of personal information. Published results will not contain any personal data and individuals will not be identifiable. All trial related data will be archived for five years.

Study Dissemination
Ownership of the data arising from this study resides with the study team. The primary trial publication will be drafted by a writing committee and will recognise the work of those involved in the trial. PIs and investigators on delegation logs for patient recruitment will be invited as collaborators on the primary trial publication. Trial results will be published in a peer-reviewed journal, presented at meetings and published as a plain language summary and visual abstract.

Study Registration
Registered at ClinicalTrials.gov (NCT04895644)

AUTHORS' CONTRIBUTIONS
All authors contributed to the conception and design of the work, drafting the work and revising it critically for important intellectual content, final approval of the version to be published, and agree to be accountable for all aspects of the work in ensuring that questions related to the accuracy or integrity of any part of the work are appropriately investigated and resolved.

ACKNOWLEDGEMENTS
We thank all patients, lay members, and members of patient and public involvement groups who gave feedback to improve the study and design a study that is relevant and helpful for older or frail patients with odontoid process fractures.

FUNDING STATEMENT
This work was supported by the National Institute for Health Research (NIHR) Health Technology Assessment (HTA) grant number 131118. EE, JW, MJR, CK, JN, DJL, SDS, and PFXS are supported by this grant. The funder had no role in the study design, writing of the protocol, or the decision to submit the paper for publication.

For peer review only - http://bmjopen.bmj.com/site/about/guidelines.xhtml

COMPETING INTERESTS STATEMENT
MJR is supported by an NHS Research Scotland Career Researcher Clinician award. EE is supported by Plymouth Charitable Fund and a University of Plymouth pump priming grant. All other authors declared no competing interests.

FIGURE LEGENDS

Figure 1: DENS Trial Flowchart

NDI: neck disability index; VAS: visual analogue scale; CT: computed tomography; C-spine: cervical spine; DISH: diffuse idiopathic skeletal hyperostosis

For peer review only

For peer review only - http://bmjopen.bmj.com/site/about/guidelines.xhtml

REFERENCES

1. Iyer S, Hurlbert RJ, Albert TJ. Management of Odontoid Fractures in the Elderly: A Review of the Literature and an Evidence-Based Treatment Algorithm. *Neurosurgery* 2018;82(4):419-30. doi: 10.1093/neuros/nyx546 [published Online First: 2017/11/23]
2. Kehoe A, Smith JE, Edwards A, et al. The changing face of major trauma in the UK. *Emerg Med J* 2015;32(12):911-5. doi: 10.1136/emermed-2015-205265 [published Online First: 2015/11/26]
3. Smith HE, Kerr SM, Fehlings MG, et al. Trends in epidemiology and management of type II odontoid fractures: 20-year experience at a model system spine injury tertiary referral center. *J Spinal Disord Tech* 2010;23(8):501-5. doi: 10.1097/BSD.0b013e3181cc43c7 [published Online First: 2010/10/14]
4. Cooper Z, Mitchell SL, Lipsitz S, et al. Mortality and Readmission After Cervical Fracture from a Fall in Older Adults: Comparison with Hip Fracture Using National Medicare Data. *J Am Geriatr Soc* 2015;63(10):2036-42. doi: 10.1111/jgs.13670 [published Online First: 2015/10/13]
5. Graffeo CS, Perry A, Puffer RC, et al. Deadly falls: operative versus nonoperative management of Type II odontoid process fracture in octogenarians. *Journal of neurosurgery Spine* 2017;26(1):4-9. doi: 10.3171/2016.3.spine151202 [published Online First: 2016/08/20]
6. Morri M, Ambrosi E, Chiari P, et al. One-year mortality after hip fracture surgery and prognostic factors: a prospective cohort study. *Sci Rep* 2019;9(1):18718. doi: 10.1038/s41598-019-55196-6 [published Online First: 2019/12/12]
7. Sheikh HQ, Athanassacopoulos M, Doshi AB, et al. Early mortality and morbidity following a type II odontoid fracture in the elderly. *The surgeon : journal of the Royal Colleges of Surgeons of Edinburgh and Ireland* 2018;16(5):297-301. doi: 10.1016/j.surge.2018.03.007 [published Online First: 2018/04/17]
8. McIlroy S, Lam J, Khan MF, et al. Conservative Management of Type II Odontoid Fractures in Older People: A Retrospective Observational Comparison of Osseous Union Versus Nonunion. *Neurosurgery* 2020 doi: 10.1093/neuros/nyaa256 [published Online First: 2020/06/23]
9. Watts A, Athanassacopoulos M, Breakwell L, et al. Management of C2 Odontoid peg fragility fractures - A UK survey of spinal surgeons. British Association of Spine Surgeons Conference. Brighton, UK, 2019.
10. Woodfield J. Odontoid Fracture Survey Results (Personal communication), 2019.
11. Pal D, Sell P, Grevitt M. Type II odontoid fractures in the elderly: an evidence-based narrative review of management. *European spine journal : official publication of the European Spine Society, the European Spinal Deformity Society, and the European Section of the Cervical Spine Research Society* 2011;20(2):195-204. doi: 10.1007/s00586-010-1507-6 [published Online First: 2010/09/14]
12. Lunsford TR, Davidson M, Lunsford BR. The effectiveness of four contemporary orthoses in restricting cervical motion. *Journal of Prosthetics and Orthotics* 1994;6(4):93-99.
13. Whitcroft KL, Massouh L, Amirfeyz R, et al. A comparison of neck movement in the soft cervical collar and rigid cervical brace in healthy subjects. *Journal of manipulative and physiological therapeutics* 2011;34(2):119-22. doi: 10.1016/j.jmpt.2010.12.007 [published Online First: 2011/02/22]

For peer review only - http://bmjopen.bmj.com/site/about/guidelines.xhtml

For peer review only

14. De Bonis P, Iaccarino C, Musio A, et al. Functional Outcome of Elderly Patients Treated for Odontoid Fracture: A Multicenter Study. *Spine* 2019;44(13):951-58. doi: 10.1097/brs.0000000000002982 [published Online First: 2019/06/18]
15. Fehlings MG, Arun R, Vaccaro AR, et al. Predictors of treatment outcomes in geriatric patients with odontoid fractures: AOSpine North America multi-centre prospective GOF study. *Spine* 2013;38(11):881-6. doi: 10.1097/BRS.0b013e31828314ee [published Online First: 2013/03/06]
16. Graffeo CS, Perry A, Puffer RC, et al. Odontoid Fractures and the Silver Tsunami: Evidence and Practice in the Very Elderly. *Neurosurgery* 2016;63 Suppl 1:113-17. doi: 10.1227/neu.0000000000001279 [published Online First: 2016/07/12]
17. Harrop JS, Hart R, Anderson PA. Optimal treatment for odontoid fractures in the elderly. *Spine* 2010;35(21 Suppl):S219-27. doi: 10.1097/BRS.0b013e3181f32716 [published Online First: 2010/10/15]
18. Smith HE, Kerr SM, Maltenfort M, et al. Early complications of surgical versus conservative treatment of isolated type II odontoid fractures in octogenarians: a retrospective cohort study. *J Spinal Disord Tech* 2008;21(8):535-9. doi: 10.1097/BSD.0b013e318163570b [published Online First: 2008/12/06]
19. Lofrese G, Musio A, De Iure F, et al. Type II odontoid fracture in elderly patients treated conservatively: is fracture healing the goal? *European spine journal : official publication of the European Spine Society, the European Spinal Deformity Society, and the European Section of the Cervical Spine Research Society* 2019;28(5):1064-71. doi: 10.1007/s00586-019-05898-2 [published Online First: 2019/01/24]
20. Perry A, Graffeo CS, Carlstrom LP, et al. Fusion, Failure, Fatality: Long-term Outcomes After Surgical Versus Nonoperative Management of Type II Odontoid Fracture in Octogenarians. *World neurosurgery* 2018;110:e484-e89. doi: 10.1016/j.wneu.2017.11.020 [published Online First: 2017/11/21]
21. Crockard HA, Heilman AE, Stevens JM. Progressive myelopathy secondary to odontoid fractures: clinical, radiological, and surgical features. *J Neurosurg* 1993;78(4):579-86. doi: 10.3171/jns.1993.78.4.0579 [published Online First: 1993/04/01]
22. Peck GE, Shipway DJH, Tsang K, et al. Cervical spine immobilisation in the elderly: a literature review. *British journal of neurosurgery* 2018;32(3):286-90. doi: 10.1080/02688697.2018.1445828 [published Online First: 2018/03/01]
23. Rockwood K, Song X, MacKnight C, et al. A global clinical measure of fitness and frailty in elderly people. *CMAJ* 2005;173(5):489-95. doi: 10.1503/cmaj.050051
24. Anderson LD, D'Alonzo RT. Fractures of the odontoid process of the axis. *The Journal of bone and joint surgery American volume* 1974;56(8):1663-74. [published Online First: 1974/12/01]
25. Hopewell S, Adedire O, Copsey BJ, et al. Multifactorial and multiple component interventions for preventing falls in older people living in the community. *The Cochrane database of systematic reviews* 2018;7:Cd012221. doi: 10.1002/14651858.CD012221.pub2 [published Online First: 2018/07/24]
26. Rubenstein LZ. Falls in older people: epidemiology, risk factors and strategies for prevention. *Age Ageing* 2006;35 Suppl 2:ii37-ii41. doi: 10.1093/ageing/afl084 [published Online First: 2006/08/24]
27. Benish BM, Smith KJ, Schwartz MH. Validation of a miniature thermochron for monitoring thoracolumbosacral orthosis wear time. *Spine* 2012;37(4):309-15. doi: 10.1097/BRS.0b013e31821e1488 [published Online First: 2011/05/05]

For peer review only - http://bmjopen.bmj.com/site/about/guidelines.xhtml

28. Posch M, Klinglmueller F, König F, et al. Estimation after blinded sample size reassessment. *Stat Methods Med Res* 2018;27(6):1830-46. doi: 10.1177/0962280216670424 [published Online First: 2016/10/05]
29. Frison L, Pocock SJ. Repeated measures in clinical trials: analysis using mean summary statistics and its implications for design. *Stat Med* 1992;11(13):1685-704. doi: 10.1002/sim.4780111304 [published Online First: 1992/09/30]
30. Breckenridge JP, Gianfrancesco C, de Zoysa N, et al. Mobilising knowledge between practitioners and researchers to iteratively refine a complex intervention (DAFNEplus) pre-trial: protocol for a structured, collaborative working group process. *Pilot Feasibility Stud* 2018;4:120. doi: 10.1186/s40814-018-0314-4 [published Online First: 2018/07/11]
31. NICE. Guide to the methods of technology appraisal. http://wwwniceorguk/article/pmg9/resources/non-guidance-guide-to-the-methods-of-technology-appraisal-2013-pdf, 2013.
32. Glick HA, Doshi JA, Sonnad AA, et al. Economic Evaluation in Clinical Trials. Oxford: Oxford University Press 2007.
33. Curtis L, Burns A. Unit Costs of Health and Social Care 2019. Canterbury: Personal Social Services Reseach Unit, 2018.
34. Department of Health. The Reference Costs Publications, 2019.

For peer review only

For peer review only - http://bmjopen.bmj.com/site/about/guidelines.xhtml

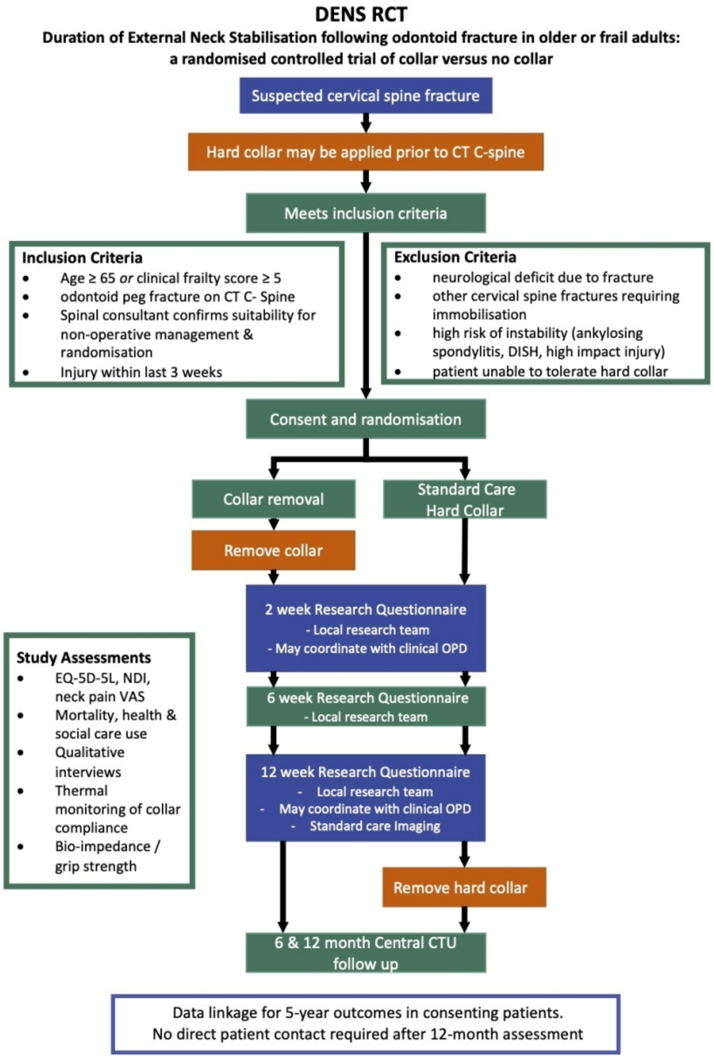

Figure 1: DENS Trial Flowchart

NDI: neck disability index; VAS: visual analogue scale; CT: computed tomography; C-spine: cervical spine; DISH: diffuse idiopathic skeletal hyperostosis

419x594mm (72 x 72 DPI)

STANDARD PROTOCOL ITEMS: RECOMMENDATIONS FOR INTERVENTIONAL TRIALS

SPIRIT 2013 Checklist: Recommended items to address in a clinical trial protocol and related documents*

Section/item	Item No	Description	Page
**Administrative information**			
Title	1	Descriptive title identifying the study design, population, interventions, and, if applicable, trial acronym	1
Trial registration	2a	Trial identifier and registry name. If not yet registered, name of intended registry	2
	2b	All items from the World Health Organization Trial Registration Data Set	1-16
Protocol version	3	Date and version identifier	1
Funding	4	Sources and types of financial, material, and other support	12
Roles and responsibilities	5a	Names, affiliations, and roles of protocol contributors	1
	5b	Name and contact information for the trial sponsor	12
	5c	Role of study sponsor and funders, if any, in study design; collection, management, analysis, and interpretation of data; writing of the report; and the decision to submit the report for publication, including whether they will have ultimate authority over any of these activities	12
	5d	Composition, roles, and responsibilities of the coordinating centre, steering committee, endpoint adjudication committee, data management team, and other individuals or groups overseeing the trial, if applicable (see Item 21a for data monitoring committee)	11-12
**Introduction**			
Background and rationale	6a	Description of research question and justification for undertaking the trial, including summary of relevant studies (published and unpublished) examining benefits and harms for each intervention	4
	6b	Explanation for choice of comparators	4
Objectives	7	Specific objectives or hypotheses	4
Trial design	8	Description of trial design including type of trial (eg, parallel group, crossover, factorial, single group), allocation ratio, and framework (eg, superiority, equivalence, noninferiority, exploratory)	5-10

For peer review only - http://bmjopen.bmj.com/site/about/guidelines.xhtml

Methods: Participants, interventions, and outcomes

Study setting	9	Description of study settings (eg, community clinic, academic hospital) and list of countries where data will be collected. Reference to where list of study sites can be obtained	5-6
Eligibility criteria	10	Inclusion and exclusion criteria for participants. If applicable, eligibility criteria for study centres and individuals who will perform the interventions (eg, surgeons, psychotherapists)	5
Interventions	11a	Interventions for each group with sufficient detail to allow replication, including how and when they will be administered	6
	11b	Criteria for discontinuing or modifying allocated interventions for a given trial participant (eg, drug dose change in response to harms, participant request, or improving/worsening disease)	6
	11c	Strategies to improve adherence to intervention protocols, and any procedures for monitoring adherence (eg, drug tablet return, laboratory tests)	7
	11d	Relevant concomitant care and interventions that are permitted or prohibited during the trial	6-8
Outcomes	12	Primary, secondary, and other outcomes, including the specific measurement variable (eg, systolic blood pressure), analysis metric (eg, change from baseline, final value, time to event), method of aggregation (eg, median, proportion), and time point for each outcome. Explanation of the clinical relevance of chosen efficacy and harm outcomes is strongly recommended	6
Participant timeline	13	Time schedule of enrolment, interventions (including any run-ins and washouts), assessments, and visits for participants. A schematic diagram is highly recommended (see Figure)	Table 1 Figure 1
Sample size	14	Estimated number of participants needed to achieve study objectives and how it was determined, including clinical and statistical assumptions supporting any sample size calculations	8
Recruitment	15	Strategies for achieving adequate participant enrolment to reach target sample size	8

Methods: Assignment of interventions (for controlled trials)

Allocation:

Sequence generation	16a	Method of generating the allocation sequence (eg, computer-generated random numbers), and list of any factors for stratification. To reduce predictability of a random sequence, details of any planned restriction (eg, blocking) should be provided in a separate document that is unavailable to those who enrol participants or assign interventions	6

Allocation concealment mechanism	16b	Mechanism of implementing the allocation sequence (eg, central telephone; sequentially numbered, opaque, sealed envelopes), describing any steps to conceal the sequence until interventions are assigned	6
Implementation	16c	Who will generate the allocation sequence, who will enrol participants, and who will assign participants to interventions	6
Blinding (masking)	17a	Who will be blinded after assignment to interventions (eg, trial participants, care providers, outcome assessors, data analysts), and how	6
	17b	If blinded, circumstances under which unblinding is permissible, and procedure for revealing a participant's allocated intervention during the trial	6
**Methods: Data collection, management, and analysis**			
Data collection methods	18a	Plans for assessment and collection of outcome, baseline, and other trial data, including any related processes to promote data quality (eg, duplicate measurements, training of assessors) and a description of study instruments (eg, questionnaires, laboratory tests) along with their reliability and validity, if known. Reference to where data collection forms can be found, if not in the protocol	Table 1 Figure 1
	18b	Plans to promote participant retention and complete follow-up, including list of any outcome data to be collected for participants who discontinue or deviate from intervention protocols	6,7,11
Data management	19	Plans for data entry, coding, security, and storage, including any related processes to promote data quality (eg, double data entry; range checks for data values). Reference to where details of data management procedures can be found, if not in the protocol	11-12
Statistical methods	20a	Statistical methods for analysing primary and secondary outcomes. Reference to where other details of the statistical analysis plan can be found, if not in the protocol	9-10
	20b	Methods for any additional analyses (eg, subgroup and adjusted analyses)	9-10
	20c	Definition of analysis population relating to protocol non-adherence (eg, as randomised analysis), and any statistical methods to handle missing data (eg, multiple imputation)	9-10
**Methods: Monitoring**			
Data monitoring	21a	Composition of data monitoring committee (DMC); summary of its role and reporting structure; statement of whether it is independent from the sponsor and competing interests; and reference to where further details about its charter can be found, if not in the protocol. Alternatively, an explanation of why a DMC is not needed	11

For peer review only - http://bmjopen.bmj.com/site/about/guidelines.xhtml

	21b	Description of any interim analyses and stopping guidelines, including who will have access to these interim results and make the final decision to terminate the trial	7-8 Table 2
Harms	22	Plans for collecting, assessing, reporting, and managing solicited and spontaneously reported adverse events and other unintended effects of trial interventions or trial conduct	11
Auditing	23	Frequency and procedures for auditing trial conduct, if any, and whether the process will be independent from investigators and the sponsor	11
**Ethics and dissemination**			
Research ethics approval	24	Plans for seeking research ethics committee/institutional review board (REC/IRB) approval	10
Protocol amendments	25	Plans for communicating important protocol modifications (eg, changes to eligibility criteria, outcomes, analyses) to relevant parties (eg, investigators, REC/IRBs, trial participants, trial registries, journals, regulators)	12
Consent or assent	26a	Who will obtain informed consent or assent from potential trial participants or authorised surrogates, and how (see Item 32)	10-11
	26b	Additional consent provisions for collection and use of participant data and biological specimens in ancillary studies, if applicable	N/A
Confidentiality	27	How personal information about potential and enrolled participants will be collected, shared, and maintained in order to protect confidentiality before, during, and after the trial	11-12
Declaration of interests	28	Financial and other competing interests for principal investigators for the overall trial and each study site	13
Access to data	29	Statement of who will have access to the final trial dataset, and disclosure of contractual agreements that limit such access for investigators	11-12
Ancillary and post-trial care	30	Provisions, if any, for ancillary and post-trial care, and for compensation to those who suffer harm from trial participation	N/A
Dissemination policy	31a	Plans for investigators and sponsor to communicate trial results to participants, healthcare professionals, the public, and other relevant groups (eg, via publication, reporting in results databases, or other data sharing arrangements), including any publication restrictions	12
	31b	Authorship eligibility guidelines and any intended use of professional writers	12
	31c	Plans, if any, for granting public access to the full protocol, participant-level dataset, and statistical code	12

For peer review only - http://bmjopen.bmj.com/site/about/guidelines.xhtml

Appendices

Informed consent materials	32	Model consent form and other related documentation given to participants and authorised surrogates
Biological specimens	33	Plans for collection, laboratory evaluation, and storage of biological specimens for genetic or molecular analysis in the current trial and for future use in ancillary studies, if applicable

*It is strongly recommended that this checklist be read in conjunction with the SPIRIT 2013 Explanation & Elaboration for important clarification on the items. Amendments to the protocol should be tracked and dated. The SPIRIT checklist is copyrighted by the SPIRIT Group under the Creative Commons "Attribution-NonCommercial-NoDerivs 3.0 Unported" license.

For peer review only

BMJ Open

Duration of External Neck Stabilisation (DENS) following odontoid fracture in older or frail adults: protocol for a randomised controlled trial of collar versus no collar

Journal:	*BMJ Open*
Manuscript ID	bmjopen-2021-057753.R1
Article Type:	Protocol
Date Submitted by the Author:	27-Mar-2022
Complete List of Authors:	Woodfield, Julie; NHS Lothian, Department of Clinical Neurosciences; The University of Edinburgh Centre for Clinical Brain Sciences, Translational Neurosurgery<br>Edlmann, Ellie; Derriford Hospital, Southwest Neurosurgical Centre; University of Plymouth, Peninsula Medical School<br>Black, Polly; NHS Lothian, Emergency Medicine Research Group (EMERGE)<br>Boyd, Julia; University of Edinburgh, Edinburgh Clinical Trials Unit<br>Copley, Phillip; NHS Lothian, Department of Clinical Neurosciences<br>Cranswick, Gina; University of Edinburgh , Edinburgh Clinical Trials Unit<br>Eborall, Helen; University of Edinburgh , Usher Institute<br>Keerie, Catriona; University of Edinburgh College of Medicine and Veterinary Medicine, Edinburgh Clinical Trials Unit<br>Khan, Sadaquate; NHS Lothian, Department of Clinical Neurosciences<br>Lawton, Julia; University of Edinburgh, Centre for Population Health Sciences<br>Lowe, David; Queen Elizabeth University Hospital Campus, Emergency Departmebnt; University of Glasgow Institute of Health and Wellbeing<br>Norrie, John; Edinburgh Clinical Trials Unit, University of Edinburgh No. 9, Bioquarter<br>Niven, Angela; Edinburgh Clinical Trials Unit<br>Reed, Matthew; Royal Infirmary of Edinburgh, Emergency Medicine Research Group; The University of Edinburgh Usher Institute of Population Health Sciences and Informatics<br>Shenkin, Susan; University of Edinburgh College of Medicine and Veterinary Medicine, Advanced Care Research Centre; The University of Edinburgh Usher Institute of Population Health Sciences and Informatics<br>Statham, Patrick; NHS Lothian, Department of Clinical Neurosciences<br>Stoddart, Andrew; The University Of Edinburgh, Edinburgh Health Services Research Unit<br>Tomlinson, James; Sheffield Teaching Hospitals NHS Foundation Trust<br>Brennan, Paul; The University of Edinburgh Centre for Clinical Brain Sciences, Translational Neurosurgery; NHS Lothian, Department of Clinical Neurosciences
**Primary Subject Heading**:	Geriatric medicine
Secondary Subject Heading:	Evidence based practice

Keywords:	Protocols & guidelines < HEALTH SERVICES ADMINISTRATION & MANAGEMENT, NEUROSURGERY, ORTHOPAEDIC & TRAUMA SURGERY

SCHOLARONE™
Manuscripts

Title

Duration of External Neck Stabilisation (DENS) following odontoid fracture in older or frail adults: protocol for a randomised controlled trial of collar versus no collar

Authors

Julie Woodfield,[1,2] Ellie Edlmann,[3,4] Polly Black,[5] Julia Boyd,[6] Phillip Correia Copley,[2] Gina Cranswick,[6] Helen Eborall,[7] Catriona Keerie,[6] Sadaquate Khan,[2] Julia Lawton,[7] David J Lowe,[8,9] John Norrie,[6] Angela Niven,[6] Matthew J Reed,[5,7] Susan D Shenkin,[7,10] Patrick FX Statham,[2] Andrew Stoddart,[6] James Tomlinson,[11] Paul M Brennan[1,2]

1. Translational Neurosurgery, Centre for Clinical Brain Sciences, University of Edinburgh, Edinburgh, UK
2. Department of Clinical Neurosciences, NHS Lothian, Edinburgh, UK
3. Southwest Neurosurgical Centre, Derriford Hospital, Plymouth, UK
4. Peninsula Medical School, University of Plymouth, Plymouth, UK
5. Emergency Medicine Research Group Edinburgh (EMERGE), Department of Emergency Medicine, Royal Infirmary of Edinburgh, Edinburgh, UK
6. Edinburgh Clinical Trials Unit, Edinburgh, UK
7. Usher Institute, University of Edinburgh, Edinburgh, UK
8. Emergency Department, Queen Elizabeth University Hospital, NHS Greater Glasgow and Clyde, Glasgow, UK
9. Institute of Health and Wellbeing, University of Glasgow, Glasgow, UK
10. Advanced Care Research Centre, University of Edinburgh, Edinburgh
11. Sheffield Teaching Hospitals NHS Foundation Trust, Sheffield, UK

Corresponding Author

Mr Paul Brennan, Translational Neurosurgery, Centre for Clinical Brain Sciences, Chancellor's Building, 49 Little France Crescent, Edinburgh, EH16 4SB, United Kingdom

Paul.brennan@ed.ac.uk

Tel: +44 (0) 131 537 1000

Word Count: 3651

Abstract Word Count: 300

For peer review only - http://bmjopen.bmj.com/site/about/guidelines.xhtml

ABSTRACT

Introduction

Fractures of the odontoid process frequently result from low impact falls in frail or older adults. These are increasing in incidence and importance as the population ages. In the UK, odontoid fractures in older adults are usually managed in hard collars to immobilise the fracture and promote bony healing. However, bony healing does not always occur in older adults, and bony healing is not associated with quality of life, functional, or pain outcomes. Further, hard collars can cause complications such as skin pressure ulcers, swallowing difficulties, and difficulties with personal care. We hypothesise that management with no immobilisation may be superior to management in a hard collar for older or frail adults with odontoid fractures.

Methods & Analyses

This is the protocol for the Duration of External Neck Stabilisation (DENS) trial - a non-blinded randomised controlled trial comparing management in a hard collar with management without a collar for older (≥65 years) or frail (Rockwood clinical frailty scale ≥5) adults with a new odontoid fracture. 887 neurologically intact participants with any odontoid process fracture type will be randomised to continuing with a hard collar (standard care) or removal of the collar (intervention). The primary outcome is quality of life measured using the EQ-5D-5L at 12 weeks. Secondary outcomes include pain scores, neck disability index, health and social care use and costs, and mortality.

Ethics & Dissemination

Informed consent for participation will be sought from those able to provide it. We will also include those who lack capacity to ensure representativeness of frail and acutely unwell older adults. Results will be disseminated via scientific publication, lay summary, and visual abstract. The DENS trial received a favourable ethical opinion from the Scotland A Research Ethics Committee (21/SS/0036) and the Leeds West Research Ethics Committee (21/YH/0141).

Registration Details

Registered at ClinicalTrials.gov (NCT04895644).

For peer review only

For peer review only - http://bmjopen.bmj.com/site/about/guidelines.xhtml

Strengths & Limitations of this study

- The DENS trial is a multi-centre non-blinded randomised controlled trial comparing the use of hard collars with no immobilisation.
- The primary outcome measure (EQ-5D-5L) is a quality of life measure relevant to the older or frail population.
- Health economic analysis and embedded qualitative study methods assess the wider implications of collar use in the older or frail population.
- Inclusion of all odontoid process fracture types, frail patients, and those with cognitive impairment will ensure the results are applicable to these populations.
- Qualitative methods are used to assess and maximise trial recruitment strategies.

Key Words
Odontoid process, odontoid peg, C2, cervical fracture, axis, dens, older adults, elderly, frailty, low-impact falls, cervical immobilisation, hard collar

For peer review only

For peer review only - http://bmjopen.bmj.com/site/about/guidelines.xhtml

INTRODUCTION

Background and Rationale

Odontoid process (dens) fractures occur following low impact falls in frail and older people, and are increasing in incidence as the population ages.[1-3] The Trauma Audit and Research Network (TARN) database identified at least 1,700 odontoid fractures each year in England and Wales, 85% in people over 65 years. One-year mortality following an odontoid fracture is 20-50%, similar to or higher than following a hip fracture, likely reflecting the underlying frailty and health status of those at risk of low impact falls.[4-7]

In the UK, 85-90% of frail or older people with a new odontoid fracture are managed non-operatively, most with 6-12 weeks immobilisation in a hard collar.[8-10] This aims to promote bony fusion and prevent neurological deterioration or instability pain.[1 5 11] However, hard collars only restrict 40-50% of neck movements,[12 13] and bony fusion rates of 20-80% are variable across fracture types, management, age, and frailty.[1 5 11 14-18] Further, bony fusion may not be associated with pain, quality of life, mortality, or functional outcomes in older people,[8 19 20] and late neurological deterioration is very rare.[5 11 21] Hard collars can cause skin pressure ulcers and difficulties with swallowing, breathing, and personal care, all of which can affect quality of life (QoL).[10 22] Additional health and social care input is often required to assist patients with activities of daily living. If fibrous union, or lack of bony fusion is an acceptable outcome and hard collars can negatively impact QoL, then management of an odontoid fracture with a hard collar may be causing unnecessary harm and negatively impacting on QoL in a group for whom the short to medium term QoL may be more important than long term outcomes.

Spinal surgeons responding to a UK survey variably reported managing odontoid fractures without any immobilisation or removing hard collars in frail or older patients who were unable to tolerate a collar, had a short life expectancy, or suffered complications.[10] Ninety percent supported randomisation to management with or without a collar to determine optimum treatment.[10] Further, feedback from patients managed in collars supports exploring whether collars are necessary for odontoid process fractures.

The Duration of External Neck Stabilisation (DENS) trial will test the hypothesis that management of odontoid process fractures without immobilisation is associated with improved QoL compared to management in a hard collar for 12 weeks.

Aims and Objectives

The primary aim is to determine the difference in QoL measured using the EQ-5D-5L at 12 weeks between frail or older patients with odontoid process fractures managed with a hard collar (standard care) or without any immobilisation (intervention).

Secondary aims include assessing differences in neck pain, functional recovery, complications, radiological findings, mortality, and overall health-care use and costs, up to one year post injury.

A nested qualitative study will explore trial recruitment experiences from the perspectives of patients, carers, and healthcare professionals, and explore adherence to group allocation.

For peer review only - http://bmjopen.bmj.com/site/about/guidelines.xhtml

Findings from the qualitative study will inform recruitment and consent procedures and aid interpretation of trial results.

METHODS AND ANALYSIS

Study design

This manuscript has been prepared in accordance with the SPIRIT checklist. The DENS trial is a non-blinded randomised controlled trial with a nested qualitative study comparing early hard collar removal (intervention) with treatment in a hard collar for 12 weeks (standard care) in older or frail adults with a new odontoid process fracture. Figure 1 shows the trial flow chart. The study will recruit from November 2021 to December 2023.

Study Eligibility

Inclusion Criteria:

- Aged ≥65 years, or Rockwood clinical frailty scale (CFS) of ≥5 (at least mildly frail: help needed in high order instrumental activities of daily living)[23]
- Recent odontoid process fracture of any type[24] secondary to low impact trauma (any degree of fracture angulation, displacement or canal narrowing)
- History of recent trauma (within 3 weeks)
- Recruited within 3 weeks of injury
- Determined by consultant spinal surgeon as suitable for standard treatment in hard collar or treatment without a collar

Exclusion Criteria:

- Fracture sustained in a high-impact injury
- New neurological deficit attributable to the fracture
- Unable to tolerate a hard collar
- Additional (non-odontoid process) cervical spine fracture not suitable for management without a hard collar
- Underlying condition with risk of spinal instability (e.g. ankylosing spondylitis)
- Fracture suspected to be older than three weeks at the time of assessment
- Not expected to survive to hospital discharge

A consultant radiologist report of an odontoid process fracture on CT and confirmation of eligibility by a consultant spinal surgeon (orthopaedic or neurosurgical) is required. Patients with additional cervical spine fractures are eligible provided the consultant spinal surgeon determines the fracture(s) are suitable for management without a hard collar. There are no exclusions for other injuries (e.g. fractured femur). Co-enrolment in other studies is permitted where this does not affect the DENS trial randomisation allocation or outcome measure assessment, and where this is not expected to burden the participant.

Participant Recruitment

Potential participants will be identified by clinicians following presentation to Emergency Departments (EDs) or acute assessment units, through review of acute cervical spine CT reports, acute hospital attendance, or through referrals or admissions to spinal services in the UK. An anonymised screening log will be kept.

For peer review only - http://bmjopen.bmj.com/site/about/guidelines.xhtml

Any neck stabilisation can be applied as part of standard care prior to randomisation, including; trauma collar, blocks, spinal board, padded hard collar (e.g. Miami J, Aspen, Philadelphia) or soft collar. Standard care treatment, such as removal from spinal boards and placement of a padded hard collar will occur as usual, and will not be delayed by trial assessment and participation.

Eligibility assessment and randomisation will take place as soon as possible after injury (target within 48 hours) to maximise the impact of study intervention. However, falls in older and frail people are usually multifactorial,[25 26] and conditions leading to the fall or resulting from the fall may affect capacity and fitness to participate. Recruitment can occur up to 3 weeks after the injury to provide flexibility for those presenting late, facilitate participation in those who are acutely unwell, and ensure time for involvement of patient representatives where needed.

The nested qualitative study will include healthcare professionals involved in patient recruitment, study participants, carers of participants lacking capacity, and those who decline trial participation.

Randomisation and Interventions
Web-based randomisation will occur with a minimisation algorithm based on:

- Age at randomisation (<75 versus ≥75 years)
- Odontoid Fracture type (I-III)[24]
- Frailty (CFS ≥5 versus <5)[23]

The first participant will be allocated to one arm with a probability of 0.5 and subsequent participants will be allocated with a probability of 0.8 to the group which minimises differences of the above variables between the two trial arms. Treatment allocation will be disclosed to the local research team via the web interface following randomisation.

The standard care arm will involve 12 weeks of hard collar treatment, following local usual choice of hard collar and local usual arrangements for collar care. For those allocated to the intervention (collar removal) arm, the collar will be removed as soon as possible, but may be weaned over several days if required for optimising analgesia. Removal of a collar prior to 12 weeks in the hard collar arm or wearing of a collar in the no collar arm will be recorded as a failure of adherence with explanation. Soft collars will not be used in either arm.

Outcome Measures
The primary outcome measure is the EQ-5D-5L at 12 weeks post-randomisation.
Secondary outcome measures are:

- 2 & 6 weeks: EQ-5D-5L, neck disability index (NDI), neck numeric pain scale
- 12 weeks: NDI, neck numeric pain scale, adverse events (AEs), compliance with hard collar use, analgesia use, length of hospital stay, discharge destination, bony fusion / stability on imaging, loss of muscle bulk in upper limbs using grip strength and bio-impedence

For peer review only - http://bmjopen.bmj.com/site/about/guidelines.xhtml

- 6 & 12 months: EQ-5D-5L, NDI, neck numeric pain scale, injury-related complications, mortality, health and social care visits/use, hospital admission or outpatient visits, health economics analysis

Trial assessments

Assessments and timing of data collection are shown in Table 1. Baseline data on demographics, comorbidities, injury, radiological findings and inpatient management will be recorded. Patient diaries will be used to collect pain scores, analgesia and collar use. Standard local clinical follow up will occur. Research assessments will be carried out at 2, 6 and 12 weeks by the local research team and by a blinded member of the central research team at 6 and 12 months. Assessments will occur via telephone interviews, postal or online questionnaires, or during standard care appointments. A proxy can provide information on a patient's behalf when required. Up to three reminder telephone calls will be made to maximise completion.

For peer review only

Table 1: **Timing of Study Assessments.**

Assessments undertaken as standard care are shown with x. Study specific assessments are shown with *. Assessments may occur within the following time windows: 2 weeks (±1 week), 6 and 12 weeks (± 2 weeks) and 6 and 12 months (± 4 weeks). Fracture type refers to that of Anderson & D'Alonzo.[24] (CFS: Rockwood clinical frailty scale;[23] NDI: neck disability index; AEs: adverse events)

	**Timing from Randomisation**						
	Baseline	Discharge	2 weeks	6 weeks	12 weeks	6 months	12 months
**Assessment**							
Eligibility Assessment	*						
Consent	*						
**Demographics**							
Baseline data	*						
CFS	*						
**Imaging**							
Fracture Type	x						
Imaging Type	x				x		
**Outcomes**							
EQ-5D-5L			*	*	*	*	*
EQ-VAS			*	*	*	*	*
Neck numeric pain scale			*	*	*	*	*
NDI			*	*	*	*	*
AEs		*			*		
Injury-related complications						*	*
Collar use		*	*	*	*		
Grip strength/bioimpedance	*				*		
Service use			*	*	*	*	*
Analgesia use			*	*	*		
Mortality					*	*	*

At centres with facilities for routine assessment of grip strength and bio-impedence, all participants will be invited to take part in this arm of the study. Loss of muscle bulk in the

upper limbs will be assessed over 12 weeks using hand grip strength measured with a dynamometer.[27] Bio-impedence measurements will be made at the wrist and ankle. Muscle mass and skeletal muscle index as a marker of frailty will be calculated as per Janssen et al.[28]

Approximately 25 patients in the collar arm will use temperature sensors to assess compliance with the collar (Thermochron iButtons).[29] These will be the first participants willing to participate at centres where the temperature sensors can be missioned. Long term follow up of mortality and hospital admissions using anonymised data without participant contact will occur for 5 years.

No study specific follow up imaging is mandatory. Participants will follow local clinical protocols for imaging. The modality and findings of all imaging undertaken will be recorded and imaging collected at the study centre for analysis where patient consent for transfer is given.

Qualitative study
Qualitative researchers will interview healthcare professionals about their recruitment experiences, perceptions of why patients decline or consent to participation, and reasons for not approaching eligible patients. Patients and caregivers will be interviewed at two time points – following randomisation and 12 weeks later. Post-randomisation interviews will explore reasons for taking part or declining participation, views about trial recruitment approaches, hopes and expectations regarding trial participation, and any anticipated difficulties or concerns adhering to treatment allocation. Follow-up interviews will explore treatment adherence, benefits or burdens of the allocated treatment, and perceptions of the impact on QoL and recovery. All interviews will use topic guides to help ensure the discussion remains relevant to addressing the study aims, while offering participants flexibility to raise issues they consider important, including those unforeseen at the study outset.

Sample Size
The recruitment target is 887 participants from around 25 UK sites over 2 years. An unadjusted sample size was calculated based on 90% power and a 5% significance level, using a two-sided two-sample t-test, for an effect size of 1/6 (0.05 minimum clinically important difference / 0.3 standard deviation) on the EQ-5D-5L at 12 weeks. This results in a sample size of 1514, increasing to 1893 assuming 20% missing data. This sample size can be reduced by accounting for correlation between baseline covariates (age, CFS) and 12 week EQ-5D-5L. It is anticipated that this correlation will be around 0.5, allowing a 25% reduction in sample size. Higher correlation will allow a larger reduction.[30] Further, the post-randomisation measures of EQ-5D-5L at 2, 6 and 12 weeks, are assumed to have a serial correlation of 0.44 which allows a further 37.5% reduction in sample size.[30 31] Including these reductions leads to the final target sample size of 887, with 1:1 allocation.

To check these assumptions, a sample size re-estimation will be modelled using a simulation approach based on 12 week outcome data from the first 300 participants.[30] Due to the conservative assumptions made, particularly the assumed standard deviation, the probability of increasing the sample size is small, and there is a larger probability it can be reduced.

For peer review only - http://bmjopen.bmj.com/site/about/guidelines.xhtml
For peer review only

Based on audits in two UK neurosurgical units, and assuming a 50% recruitment rate with staggered site opening, the target recruitment rate is 1.6 patients per site per month. An internal pilot will assess recruitment feasibility, targeting 132 patients (15% total) across 18 centres in 9 months. Progression criteria for the pilot are shown in Table 2.

Table 2: Study Progression Criteria

Study progression criteria for the internal pilot to assess recruitment feasibility after the first 9 months of recruitment. The traffic light categories will lead to the following actions. Green: continue unchanged. Amber: make changes, including adding more sites or increasing the time period for recruitment. Red: continue stopping as study may not feasible, unless identifiable and rectifiable causes are identified. Adherence failure includes study withdrawals and cross-over (removing a collar in the collar arm, or wearing a collar in the no collar arm).

	Category		
	Red	Amber	Green
Total number recruited	≤108	109-131	≥132
Recruitment threshold (%)	≤82%	83%-99%	100%
Recruitment rate (patients per site per month)	1.07	1.33	1.63
Number of sites open	<5	5-10	>10
Adherence failure	>20%	11-20%	≤10%

The qualitative study will recruit approximately 20 healthcare professionals and 30 patients or carers from a cross section of sites. Purposive sampling will be used to ensure representation of different ages, socio-economic backgrounds, cognitions, and residential setting amongst patients. Sampling decisions may be revised in light of emerging findings.

Data Analysis Plan

The primary data analysis will be by intention to treat. A 5% two-sided significance level will be used throughout. A detailed statistical analysis plan will be developed by the study statisticians and finalised prior to locking of the trial database.

Analysis of the primary outcome will be a repeated measures analysis of covariance, including terms for treatment arm and the EQ-5D-5L responses at 2, 6 and 12 weeks. Adjustments will be made for the variables included in the randomisation minimisation algorithm. Adjustment for study site will be included as a random effect, if appropriate. The repeated measures approach enables estimation of an intervention effect at week 12 post-randomisation (primary outcome), and an overall assessment of the effect of the intervention over the 12-week period.

For peer review only - http://bmjopen.bmj.com/site/about/guidelines.xhtml

For peer review only

The 12-week NDI and VAS will be analysed in a similar manner to the EQ-5D-5L. It is anticipated that results will underpin any between group differences seen in EQ-5D-5L, confirming and strengthening the clinical interpretation of the findings. All other secondary outcomes will be analysed with statistical models appropriate to the distribution of the outcome. Where there are data recorded on multiple occasions post randomisation we may use an appropriate repeated measures model. Exploratory subgroup analyses by fracture type (I, II and III),[24] CFS (<5 versus ≥5)[23] and age (<75 versus ≥ 75 years) will be performed. The influence of any missing data on the robustness of the findings will be examined e.g. using multiple imputation models under a missing at random assumption. Screening logs will be analysed to assess generalisability of results.

Interview data will be analysed thematically using the method of constant comparison. To ensure rigour, several individuals will be involved in data analysis and coding. An interactive working group will be set up, comprising the qualitative research team, co-investigators, the trial manager, PPI representatives, and at least one healthcare professional from each of the sites involved in the pilot phase. A 'what, so what, now what' approach[32] will be used to translate qualitative findings and other experiences shared by group members into tangible recommendations.

A 12 month within trial health economic analysis will be undertaken based on National Institute for Health and Care Excellence (NICE) reference case recommendations to maximise UK policy relevance.[33] This will include: adoption of a National Health Service (NHS) and Personal Social Services decision perspective; cost-utility approach for primary analysis (results presented in terms of incremental cost per quality adjusted life year (QALY), derived from EQ-5D-5L data with an area under the curve approach, omitting baseline); discount rate of 3.5% for both costs and QALYs (where applicable); and use of probabilistic sensitivity analysis, to generate cost effectiveness acceptability curves via the recycled predictions technique.[33 34] Choice of primary analysis cost per QALY threshold and EQ-5D-5L scoring algorithm will match NICE preferences at the time of data lock. Resource use will be combined with standard UK price weights to generate costs.[35 36] The latest financial year for which at least one study participant provides data and prices are available will be selected as base year. Univariate mean QALYs, resource use, and costs will be presented for each trial arm alongside differences in means and associated 95% confidence intervals. Multivariate analysis of both QALYs and total costs, will also be presented controlling for baseline costs where available, and minimisation variables. Missing data will be imputed using appropriate techniques depending on degree of missingness, likely multiple imputation by chained equations.

Patient and Public Involvement (PPI)

Patients and the public have played an active role in developing the grant application and project protocol, are co-applicants on the grant and are represented on the Trial Steering Committee. We asked older people with odontoid fractures and members of a PPI group for their opinion on EQ-5D-5L and NDI as possible primary outcome measures. Older patients with odontoid fractures managed to complete the EQ-5D-5L, whilst some struggled with the NDI, and described some of the NDI domains as irrelevant. PPI input identified frailty rather than age as more influential of daily function, leading to inclusion criteria including both. A lay group also reviewed and rewrote the lay summary of the trial and had input into its likely

For peer review only - http://bmjopen.bmj.com/site/about/guidelines.xhtml

acceptability. At the end of the study, we will produce a plain language summary of the study results and a visual abstract, in conjunction with participants and our PPI partners.

ETHICS AND DISSEMINATION

The DENS trial received a favourable ethical opinion from the Scotland A Research Ethics Committee (REC) on the 10th June 2021 (21/SS/0036) and the Leeds West REC (on behalf of England and Wales) on the 29th July 2021 (21/YH/0141).

Participant Capacity and Consent

The study member obtaining consent will explain and clarify all relevant information, either face-to-face, or via telephone or video call, and give patients the time they need to decide whether to participate. Informed consent will be signed and dated by participants or their representative, and a study team member. Verbal consent from a participant or their representative, signed and witnessed by a study team member will also be valid. Participants may withdraw consent at any time without reason, without affecting their care.

Patients diagnosed with odontoid fractures may lack capacity. Recruitment of these patients is necessary to aid generalisability of the trial outcome and to allow equal access to any benefits of participation. All consent procedures will adhere to the Adults with Incapacity (Scotland) Act 2000, the 2005 Mental Capacity Act, the Mental Capacity Act (Northern Ireland) 2016, and their codes of practice. For patients in Scotland who lack capacity, the patient's nearest relative, welfare attorney or guardian can provide consent. For patients in England, Wales and Northern Ireland who lack capacity, a personal consultee (relative or close friend) can provide consent. Where possible this will be written and signed, otherwise clear documentation of the discussion and named persons involved will be accepted.

If a participant without capacity regains capacity, informed consent will be sought to continue in the trial. In Scotland, if a participant has given informed consent, but loses capacity, they will remain in the study. In England, Wales, and Northern Ireland, if a participant has already given informed consent, but loses capacity, they will remain in the study only if consent is then given by a consultee.

Safety Considerations

Adverse Events (AEs) will be recorded up to 12-weeks. Expected AEs relating to hard collar use or odontoid process fractures include skin pressure ulcers, dysphagia, and recurrent falls. All AEs (expected or not) will be reviewed by the local principal investigator and reported to the data monitoring committee (DMC). Serious Adverse Events (SAEs) will also be reported to the trial sponsor, who will notify the REC. Outcome data, AEs and SAEs, will be routinely reviewed by the DMC. The DMC is independent and oversees trial safety and data monitoring. If the DMC assesses that the risk-benefit balance is significantly changed by any new safety information, amendments to the trial will be made.

The trial funder (NIHR) and co-sponsors (University of Edinburgh and NHS Lothian) did not influence trial design. The sponsor ensures that data collection, management and trial monitoring are conducted appropriately and has overall responsibility for the study. Neither

For peer review only - http://bmjopen.bmj.com/site/about/guidelines.xhtml
For peer review only

the funder nor the sponsor will have ultimate authority over writing of the report or the decision to submit the report for publication.

The trial will be coordinated by the central trial team based at the Edinburgh Clinical Trials Unit (ECTU), overseen by a Trial Management Group consisting of key trial members. Regular reports will be made to the Trial Steering Committee.

The Protocol has been designed by the Chief Investigator and collaborators. The University of Edinburgh has insurance for negligent harm caused by poor protocol design, and participating sites have their own insurance and NHS indemnity for clinical negligence and other negligent harm.

Data Management

Participant records will be identified using a unique study identification number. All records will be kept in a secure storage area with limited access. Data will be entered anonymously into a secure database created and maintained by ECTU, stored on a secure server at The University of Edinburgh compliant with all relevant regulations. Trained and delegated members of the research team will have password protected logins for their own centre. Data generated from the iButton sensors is anonymised using a numerical code, which will be recorded on the database. Interviews for the qualitative study will be digitally recorded using a device encrypted to AES256 standard. Imaging will be stored and transferred within the NHS network. Only anonymised scans will be processed outside the NHS network. All investigators and study site staff will comply with the requirements of the appropriate data protection legislation (including the General Data Protection Regulation and Data Protection Act 2018) regarding collection, storage, processing and disclosure of personal information. Published results will not contain any personal data and individuals will not be identifiable. All trial related data will be archived for five years.

Study Dissemination

Ownership of the data arising from this study resides with the study team. The primary trial publication will be drafted by a writing committee and will recognise the work of those involved in the trial. PIs and investigators on delegation logs for patient recruitment will be invited as collaborators on the primary trial publication. Trial results will be published in a peer-reviewed journal, presented at meetings and published as a plain language summary and visual abstract.

Data Availability Statement

Anonymised patient-level data will be available on reasonable request to the Chief Investigator (paul.brennan@ed.ac.uk). The trial protocol and statistical analysis plans will be published and available prior to trial completion.

Study Registration

Registered at ClinicalTrials.gov (NCT04895644)

For peer review only - http://bmjopen.bmj.com/site/about/guidelines.xhtml

AUTHORS' CONTRIBUTIONS

JW, EE, PCC, PFXS, PMB contributed to the conception of the study. JW, EE, PB, JB, PCC, GC, HE, CK, SK, JL, DL, JN, AN, MJR, SDS, PFXS, AS, JT, and PBM all designed the protocol. JW, EE, JB, PCC, HE, CK, JL, DJL, MJR, SDS, and AS drafted the protocol. JW, EE, PB, JB, PCC, GC, HE, CK, SK, JL, DJL, JN, AN, MJR, SDS, PFXS, AS, JT, and PMB revised the manuscript critically for intellectual content and approved the final version for publication. PMB agrees to be accountable for all aspects of the work in ensuring that questions related to the accuracy or integrity of any part of the work are appropriately investigated and resolved.

ACKNOWLEDGEMENTS

We thank all patients, lay members, and members of patient and public involvement groups who gave feedback to improve the study and design a study that is relevant and helpful for older or frail patients with odontoid process fractures.

FUNDING STATEMENT

This work was supported by the National Institute for Health Research (NIHR) Health Technology Assessment (HTA) grant number 131118. EE, JW, MJR, CK, JN, DJL, SDS, and PFXS are supported by this grant. The funder had no role in the study design, writing of the protocol, or the decision to submit the paper for publication.

COMPETING INTERESTS STATEMENT

MJR is supported by an NHS Research Scotland Career Researcher Clinician award. EE is supported by Plymouth Charitable Fund and a University of Plymouth pump priming grant. All other authors declared no competing interests.

FIGURE LEGENDS

Figure 1: DENS Trial Flowchart

NDI: neck disability index; VAS: visual analogue scale; CT: computed tomography; C-spine: cervical spine; DISH: diffuse idiopathic skeletal hyperostosis

For peer review only

For peer review only - http://bmjopen.bmj.com/site/about/guidelines.xhtml

REFERENCES

1. Iyer S, Hurlbert RJ, Albert TJ. Management of Odontoid Fractures in the Elderly: A Review of the Literature and an Evidence-Based Treatment Algorithm. *Neurosurgery* 2018;82(4):419-30. doi: 10.1093/neuros/nyx546 [published Online First: 2017/11/23]
2. Kehoe A, Smith JE, Edwards A, et al. The changing face of major trauma in the UK. *Emerg Med J* 2015;32(12):911-5. doi: 10.1136/emermed-2015-205265 [published Online First: 2015/11/26]
3. Smith HE, Kerr SM, Fehlings MG, et al. Trends in epidemiology and management of type II odontoid fractures: 20-year experience at a model system spine injury tertiary referral center. *J Spinal Disord Tech* 2010;23(8):501-5. doi: 10.1097/BSD.0b013e3181cc43c7 [published Online First: 2010/10/14]
4. Cooper Z, Mitchell SL, Lipsitz S, et al. Mortality and Readmission After Cervical Fracture from a Fall in Older Adults: Comparison with Hip Fracture Using National Medicare Data. *J Am Geriatr Soc* 2015;63(10):2036-42. doi: 10.1111/jgs.13670 [published Online First: 2015/10/13]
5. Graffeo CS, Perry A, Puffer RC, et al. Deadly falls: operative versus nonoperative management of Type II odontoid process fracture in octogenarians. *Journal of neurosurgery Spine* 2017;26(1):4-9. doi: 10.3171/2016.3.spine151202 [published Online First: 2016/08/20]
6. Morri M, Ambrosi E, Chiari P, et al. One-year mortality after hip fracture surgery and prognostic factors: a prospective cohort study. *Sci Rep* 2019;9(1):18718. doi: 10.1038/s41598-019-55196-6 [published Online First: 2019/12/12]
7. Sheikh HQ, Athanassacopoulos M, Doshi AB, et al. Early mortality and morbidity following a type II odontoid fracture in the elderly. *The surgeon : journal of the Royal Colleges of Surgeons of Edinburgh and Ireland* 2018;16(5):297-301. doi: 10.1016/j.surge.2018.03.007 [published Online First: 2018/04/17]
8. McIlroy S, Lam J, Khan MF, et al. Conservative Management of Type II Odontoid Fractures in Older People: A Retrospective Observational Comparison of Osseous Union Versus Nonunion. *Neurosurgery* 2020 doi: 10.1093/neuros/nyaa256 [published Online First: 2020/06/23]
9. Watts A, Athanassacopoulos M, Breakwell L, et al. Management of C2 Odontoid peg fragility fractures - A UK survey of spinal surgeons. Injury 2021; doi: 10.1016/j.injury.2021.09.057
10. Woodfield J. Odontoid Fracture Survey Results (Personal communication), 2019.
11. Pal D, Sell P, Grevitt M. Type II odontoid fractures in the elderly: an evidence-based narrative review of management. *European spine journal : official publication of the European Spine Society, the European Spinal Deformity Society, and the European Section of the Cervical Spine Research Society* 2011;20(2):195-204. doi: 10.1007/s00586-010-1507-6 [published Online First: 2010/09/14]
12. Lunsford TR, Davidson M, Lunsford BR. The effectiveness of four contemporary orthoses in restricting cervical motion. *Journal of Prosthetics and Orthotics* 1994;6(4):93-99.
13. Whitcroft KL, Massouh L, Amirfeyz R, et al. A comparison of neck movement in the soft cervical collar and rigid cervical brace in healthy subjects. *Journal of manipulative*

For peer review only - http://bmjopen.bmj.com/site/about/guidelines.xhtml

and physiological therapeutics 2011;34(2):119-22. doi: 10.1016/j.jmpt.2010.12.007 [published Online First: 2011/02/22]
14. De Bonis P, Iaccarino C, Musio A, et al. Functional Outcome of Elderly Patients Treated for Odontoid Fracture: A Multicenter Study. *Spine* 2019;44(13):951-58. doi: 10.1097/brs.0000000000002982 [published Online First: 2019/06/18]
15. Fehlings MG, Arun R, Vaccaro AR, et al. Predictors of treatment outcomes in geriatric patients with odontoid fractures: AOSpine North America multi-centre prospective GOF study. *Spine* 2013;38(11):881-6. doi: 10.1097/BRS.0b013e31828314ee [published Online First: 2013/03/06]
16. Graffeo CS, Perry A, Puffer RC, et al. Odontoid Fractures and the Silver Tsunami: Evidence and Practice in the Very Elderly. *Neurosurgery* 2016;63 Suppl 1:113-17. doi: 10.1227/neu.0000000000001279 [published Online First: 2016/07/12]
17. Harrop JS, Hart R, Anderson PA. Optimal treatment for odontoid fractures in the elderly. *Spine* 2010;35(21 Suppl):S219-27. doi: 10.1097/BRS.0b013e3181f32716 [published Online First: 2010/10/15]
18. Smith HE, Kerr SM, Maltenfort M, et al. Early complications of surgical versus conservative treatment of isolated type II odontoid fractures in octogenarians: a retrospective cohort study. *J Spinal Disord Tech* 2008;21(8):535-9. doi: 10.1097/BSD.0b013e318163570b [published Online First: 2008/12/06]
19. Lofrese G, Musio A, De Iure F, et al. Type II odontoid fracture in elderly patients treated conservatively: is fracture healing the goal? *European spine journal : official publication of the European Spine Society, the European Spinal Deformity Society, and the European Section of the Cervical Spine Research Society* 2019;28(5):1064-71. doi: 10.1007/s00586-019-05898-2 [published Online First: 2019/01/24]
20. Perry A, Graffeo CS, Carlstrom LP, et al. Fusion, Failure, Fatality: Long-term Outcomes After Surgical Versus Nonoperative Management of Type II Odontoid Fracture in Octogenarians. *World neurosurgery* 2018;110:e484-e89. doi: 10.1016/j.wneu.2017.11.020 [published Online First: 2017/11/21]
21. Crockard HA, Heilman AE, Stevens JM. Progressive myelopathy secondary to odontoid fractures: clinical, radiological, and surgical features. *J Neurosurg* 1993;78(4):579-86. doi: 10.3171/jns.1993.78.4.0579 [published Online First: 1993/04/01]
22. Peck GE, Shipway DJH, Tsang K, et al. Cervical spine immobilisation in the elderly: a literature review. *British journal of neurosurgery* 2018;32(3):286-90. doi: 10.1080/02688697.2018.1445828 [published Online First: 2018/03/01]
23. Rockwood K, Song X, MacKnight C, et al. A global clinical measure of fitness and frailty in elderly people. *CMAJ* 2005;173(5):489-95. doi: 10.1503/cmaj.050051
24. Anderson LD, D'Alonzo RT. Fractures of the odontoid process of the axis. *The Journal of bone and joint surgery American volume* 1974;56(8):1663-74. [published Online First: 1974/12/01]
25. Hopewell S, Adedire O, Copsey BJ, et al. Multifactorial and multiple component interventions for preventing falls in older people living in the community. *The Cochrane database of systematic reviews* 2018;7:Cd012221. doi: 10.1002/14651858.CD012221.pub2 [published Online First: 2018/07/24]
26. Rubenstein LZ. Falls in older people: epidemiology, risk factors and strategies for prevention. *Age Ageing* 2006;35 Suppl 2:ii37-ii41. doi: 10.1093/ageing/afl084 [published Online First: 2006/08/24]

For peer review only - http://bmjopen.bmj.com/site/about/guidelines.xhtml

27. Bohannon RW. Hand-grip dynamometry predicts future outcomes in aging adults. *J Geriatr Phys Ther* 2008;31(1):3-10. doi: 10.1519/00139143-200831010-00002
28. Janssen I, Heymsfield SB, Baumgartner RN, et al. Estimation of skeletal muscle mass by bioelectrical impedance analysis. *J Appl Physiol (1985)* 2000;89(2):465-71. doi: 10.1152/jappl.2000.89.2.465
29. Benish BM, Smith KJ, Schwartz MH. Validation of a miniature thermochron for monitoring thoracolumbosacral orthosis wear time. *Spine* 2012;37(4):309-15. doi: 10.1097/BRS.0b013e31821e1488 [published Online First: 2011/05/05]
30. Posch M, Klinglmueller F, König F, et al. Estimation after blinded sample size reassessment. *Stat Methods Med Res* 2018;27(6):1830-46. doi: 10.1177/0962280216670424 [published Online First: 2016/10/05]
31. Frison L, Pocock SJ. Repeated measures in clinical trials: analysis using mean summary statistics and its implications for design. *Stat Med* 1992;11(13):1685-704. doi: 10.1002/sim.4780111304 [published Online First: 1992/09/30]
32. Breckenridge JP, Gianfrancesco C, de Zoysa N, et al. Mobilising knowledge between practitioners and researchers to iteratively refine a complex intervention (DAFNEplus) pre-trial: protocol for a structured, collaborative working group process. *Pilot Feasibility Stud* 2018;4:120. doi: 10.1186/s40814-018-0314-4 [published Online First: 2018/07/11]
33. NICE. Guide to the methods of technology appraisal. http://wwwniceorguk/article/pmg9/resources/non-guidance-guide-to-the-methods-of-technology-appraisal-2013-pdf, 2013.
34. Glick HA, Doshi JA, Sonnad AA, et al. Economic Evaluation in Clinical Trials. Oxford: Oxford University Press 2007.
35. Curtis L, Burns A. Unit Costs of Health and Social Care 2019. Canterbury: Personal Social Services Reseach Unit, 2018.
36. Department of Health. The Reference Costs Publications, 2019.

For peer review only - http://bmjopen.bmj.com/site/about/guidelines.xhtml

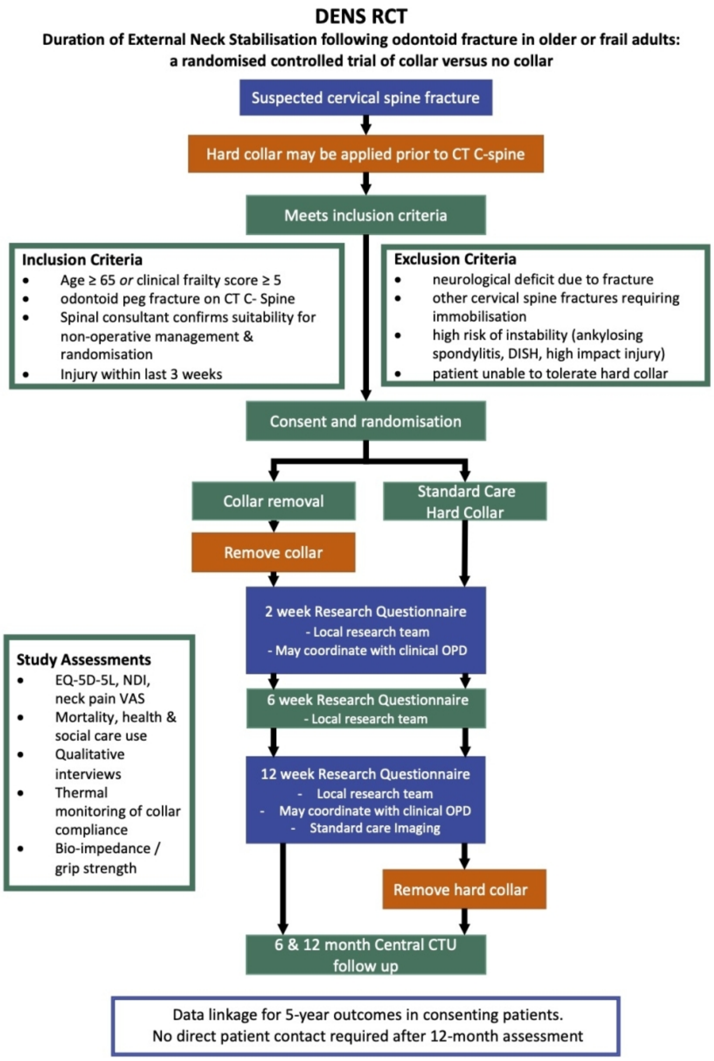

Figure 1: DENS Trial Flowchart

NDI: neck disability index; VAS: visual analogue scale; CT: computed tomography; C-spine: cervical spine; DISH: diffuse idiopathic skeletal hyperostosis

419x594mm (72 x 72 DPI)

SPIRIT

STANDARD PROTOCOL ITEMS: RECOMMENDATIONS FOR INTERVENTIONAL TRIALS

SPIRIT 2013 Checklist: Recommended items to address in a clinical trial protocol and related documents*

Section/item	Item No	Description	Page
**Administrative information**			
Title	1	Descriptive title identifying the study design, population, interventions, and, if applicable, trial acronym	1
Trial registration	2a	Trial identifier and registry name. If not yet registered, name of intended registry	2
	2b	All items from the World Health Organization Trial Registration Data Set	1-16
Protocol version	3	Date and version identifier	1
Funding	4	Sources and types of financial, material, and other support	12
Roles and responsibilities	5a	Names, affiliations, and roles of protocol contributors	1
	5b	Name and contact information for the trial sponsor	12
	5c	Role of study sponsor and funders, if any, in study design; collection, management, analysis, and interpretation of data; writing of the report; and the decision to submit the report for publication, including whether they will have ultimate authority over any of these activities	12
	5d	Composition, roles, and responsibilities of the coordinating centre, steering committee, endpoint adjudication committee, data management team, and other individuals or groups overseeing the trial, if applicable (see Item 21a for data monitoring committee)	11-12
**Introduction**			
Background and rationale	6a	Description of research question and justification for undertaking the trial, including summary of relevant studies (published and unpublished) examining benefits and harms for each intervention	4
	6b	Explanation for choice of comparators	4
Objectives	7	Specific objectives or hypotheses	4
Trial design	8	Description of trial design including type of trial (eg, parallel group, crossover, factorial, single group), allocation ratio, and framework (eg, superiority, equivalence, noninferiority, exploratory)	5-10

For peer review only

Methods: Participants, interventions, and outcomes

Study setting	9	Description of study settings (eg, community clinic, academic hospital) and list of countries where data will be collected. Reference to where list of study sites can be obtained	5-6
Eligibility criteria	10	Inclusion and exclusion criteria for participants. If applicable, eligibility criteria for study centres and individuals who will perform the interventions (eg, surgeons, psychotherapists)	5
Interventions	11a	Interventions for each group with sufficient detail to allow replication, including how and when they will be administered	6
	11b	Criteria for discontinuing or modifying allocated interventions for a given trial participant (eg, drug dose change in response to harms, participant request, or improving/worsening disease)	6
	11c	Strategies to improve adherence to intervention protocols, and any procedures for monitoring adherence (eg, drug tablet return, laboratory tests)	7
	11d	Relevant concomitant care and interventions that are permitted or prohibited during the trial	6-8
Outcomes	12	Primary, secondary, and other outcomes, including the specific measurement variable (eg, systolic blood pressure), analysis metric (eg, change from baseline, final value, time to event), method of aggregation (eg, median, proportion), and time point for each outcome. Explanation of the clinical relevance of chosen efficacy and harm outcomes is strongly recommended	6
Participant timeline	13	Time schedule of enrolment, interventions (including any run-ins and washouts), assessments, and visits for participants. A schematic diagram is highly recommended (see Figure)	Table 1 Figure 1
Sample size	14	Estimated number of participants needed to achieve study objectives and how it was determined, including clinical and statistical assumptions supporting any sample size calculations	8
Recruitment	15	Strategies for achieving adequate participant enrolment to reach target sample size	8

Methods: Assignment of interventions (for controlled trials)

Allocation:

Sequence generation	16a	Method of generating the allocation sequence (eg, computer-generated random numbers), and list of any factors for stratification. To reduce predictability of a random sequence, details of any planned restriction (eg, blocking) should be provided in a separate document that is unavailable to those who enrol participants or assign interventions	6

For peer review only - http://bmjopen.bmj.com/site/about/guidelines.xhtml

Allocation concealment mechanism	16b	Mechanism of implementing the allocation sequence (eg, central telephone; sequentially numbered, opaque, sealed envelopes), describing any steps to conceal the sequence until interventions are assigned	6
Implementation	16c	Who will generate the allocation sequence, who will enrol participants, and who will assign participants to interventions	6
Blinding (masking)	17a	Who will be blinded after assignment to interventions (eg, trial participants, care providers, outcome assessors, data analysts), and how	6
	17b	If blinded, circumstances under which unblinding is permissible, and procedure for revealing a participant's allocated intervention during the trial	6
**Methods: Data collection, management, and analysis**			
Data collection methods	18a	Plans for assessment and collection of outcome, baseline, and other trial data, including any related processes to promote data quality (eg, duplicate measurements, training of assessors) and a description of study instruments (eg, questionnaires, laboratory tests) along with their reliability and validity, if known. Reference to where data collection forms can be found, if not in the protocol	Table 1 Figure 1
	18b	Plans to promote participant retention and complete follow-up, including list of any outcome data to be collected for participants who discontinue or deviate from intervention protocols	6,7,11
Data management	19	Plans for data entry, coding, security, and storage, including any related processes to promote data quality (eg, double data entry; range checks for data values). Reference to where details of data management procedures can be found, if not in the protocol	11-12
Statistical methods	20a	Statistical methods for analysing primary and secondary outcomes. Reference to where other details of the statistical analysis plan can be found, if not in the protocol	9-10
	20b	Methods for any additional analyses (eg, subgroup and adjusted analyses)	9-10
	20c	Definition of analysis population relating to protocol non-adherence (eg, as randomised analysis), and any statistical methods to handle missing data (eg, multiple imputation)	9-10
**Methods: Monitoring**			
Data monitoring	21a	Composition of data monitoring committee (DMC); summary of its role and reporting structure; statement of whether it is independent from the sponsor and competing interests; and reference to where further details about its charter can be found, if not in the protocol. Alternatively, an explanation of why a DMC is not needed	11

For peer review only - http://bmjopen.bmj.com/site/about/guidelines.xhtml

	21b	Description of any interim analyses and stopping guidelines, including who will have access to these interim results and make the final decision to terminate the trial	7-8 Table 2
Harms	22	Plans for collecting, assessing, reporting, and managing solicited and spontaneously reported adverse events and other unintended effects of trial interventions or trial conduct	11
Auditing	23	Frequency and procedures for auditing trial conduct, if any, and whether the process will be independent from investigators and the sponsor	11
**Ethics and dissemination**			
Research ethics approval	24	Plans for seeking research ethics committee/institutional review board (REC/IRB) approval	10
Protocol amendments	25	Plans for communicating important protocol modifications (eg, changes to eligibility criteria, outcomes, analyses) to relevant parties (eg, investigators, REC/IRBs, trial participants, trial registries, journals, regulators)	12
Consent or assent	26a	Who will obtain informed consent or assent from potential trial participants or authorised surrogates, and how (see Item 32)	10-11
	26b	Additional consent provisions for collection and use of participant data and biological specimens in ancillary studies, if applicable	N/A
Confidentiality	27	How personal information about potential and enrolled participants will be collected, shared, and maintained in order to protect confidentiality before, during, and after the trial	11-12
Declaration of interests	28	Financial and other competing interests for principal investigators for the overall trial and each study site	13
Access to data	29	Statement of who will have access to the final trial dataset, and disclosure of contractual agreements that limit such access for investigators	11-12
Ancillary and post-trial care	30	Provisions, if any, for ancillary and post-trial care, and for compensation to those who suffer harm from trial participation	N/A
Dissemination policy	31a	Plans for investigators and sponsor to communicate trial results to participants, healthcare professionals, the public, and other relevant groups (eg, via publication, reporting in results databases, or other data sharing arrangements), including any publication restrictions	12
	31b	Authorship eligibility guidelines and any intended use of professional writers	12
	31c	Plans, if any, for granting public access to the full protocol, participant-level dataset, and statistical code	12

For peer review only

For peer review only - http://bmjopen.bmj.com/site/about/guidelines.xhtml

Appendices

Informed consent materials	32	Model consent form and other related documentation given to participants and authorised surrogates
Biological specimens	33	Plans for collection, laboratory evaluation, and storage of biological specimens for genetic or molecular analysis in the current trial and for future use in ancillary studies, if applicable

*It is strongly recommended that this checklist be read in conjunction with the SPIRIT 2013 Explanation & Elaboration for important clarification on the items. Amendments to the protocol should be tracked and dated. The SPIRIT checklist is copyrighted by the SPIRIT Group under the Creative Commons "Attribution-NonCommercial-NoDerivs 3.0 Unported" license.

For peer review only

Duration of External Neck Stabilisation (DENS) following odontoid fracture in older or frail adults: protocol for a randomised controlled trial of collar versus no collar

Journal:	*BMJ Open*
Manuscript ID	bmjopen-2021-057753.R2
Article Type:	Protocol
Date Submitted by the Author:	26-Jun-2022
Complete List of Authors:	Woodfield, Julie; NHS Lothian, Department of Clinical Neurosciences; The University of Edinburgh Centre for Clinical Brain Sciences, Translational Neurosurgery<br>Edlmann, Ellie; Derriford Hospital, Southwest Neurosurgical Centre; University of Plymouth, Peninsula Medical School<br>Black, Polly; NHS Lothian, Emergency Medicine Research Group (EMERGE)<br>Boyd, Julia; University of Edinburgh, Edinburgh Clinical Trials Unit<br>Copley, Phillip; NHS Lothian, Department of Clinical Neurosciences<br>Cranswick, Gina; University of Edinburgh , Edinburgh Clinical Trials Unit<br>Eborall, Helen; University of Edinburgh , Usher Institute<br>Keerie, Catriona; University of Edinburgh College of Medicine and Veterinary Medicine, Edinburgh Clinical Trials Unit<br>Khan, Sadaquate; NHS Lothian, Department of Clinical Neurosciences<br>Lawton, Julia; University of Edinburgh, Centre for Population Health Sciences<br>Lowe, David; Queen Elizabeth University Hospital Campus, Emergency Departmebnt; University of Glasgow Institute of Health and Wellbeing<br>Norrie, John; Edinburgh Clinical Trials Unit, University of Edinburgh No. 9, Bioquarter<br>Niven, Angela; Edinburgh Clinical Trials Unit<br>Reed, Matthew; Royal Infirmary of Edinburgh, Emergency Medicine Research Group; The University of Edinburgh Usher Institute of Population Health Sciences and Informatics<br>Shenkin, Susan; University of Edinburgh College of Medicine and Veterinary Medicine, Advanced Care Research Centre; The University of Edinburgh Usher Institute of Population Health Sciences and Informatics<br>Statham, Patrick; NHS Lothian, Department of Clinical Neurosciences<br>Stoddart, Andrew; The University Of Edinburgh, Edinburgh Health Services Research Unit<br>Tomlinson, James; Sheffield Teaching Hospitals NHS Foundation Trust<br>Brennan, Paul; The University of Edinburgh Centre for Clinical Brain Sciences, Translational Neurosurgery; NHS Lothian, Department of Clinical Neurosciences
**Primary Subject Heading**:	Geriatric medicine
Secondary Subject Heading:	Evidence based practice

Keywords:	Protocols & guidelines < HEALTH SERVICES ADMINISTRATION & MANAGEMENT, NEUROSURGERY, ORTHOPAEDIC & TRAUMA SURGERY

SCHOLARONE™
Manuscripts

Title

Duration of External Neck Stabilisation (DENS) following odontoid fracture in older or frail adults: protocol for a randomised controlled trial of collar versus no collar

Authors

Julie Woodfield,[1,2] Ellie Edlmann,[3,4] Polly Black,[5] Julia Boyd,[6] Phillip Correia Copley,[2] Gina Cranswick,[6] Helen Eborall,[7] Catriona Keerie,[6] Sadaquate Khan,[2] Julia Lawton,[7] David J Lowe,[8,9] John Norrie,[6] Angela Niven,[6] Matthew J Reed,[5,7] Susan D Shenkin,[7,10] Patrick FX Statham,[2] Andrew Stoddart,[6] James Tomlinson,[11] Paul M Brennan[1,2]

1. Translational Neurosurgery, Centre for Clinical Brain Sciences, University of Edinburgh, Edinburgh, UK
2. Department of Clinical Neurosciences, NHS Lothian, Edinburgh, UK
3. Southwest Neurosurgical Centre, Derriford Hospital, Plymouth, UK
4. Peninsula Medical School, University of Plymouth, Plymouth, UK
5. Emergency Medicine Research Group Edinburgh (EMERGE), Department of Emergency Medicine, Royal Infirmary of Edinburgh, Edinburgh, UK
6. Edinburgh Clinical Trials Unit, Edinburgh, UK
7. Usher Institute, University of Edinburgh, Edinburgh, UK
8. Emergency Department, Queen Elizabeth University Hospital, NHS Greater Glasgow and Clyde, Glasgow, UK
9. Institute of Health and Wellbeing, University of Glasgow, Glasgow, UK
10. Advanced Care Research Centre, University of Edinburgh, Edinburgh
11. Sheffield Teaching Hospitals NHS Foundation Trust, Sheffield, UK

Corresponding Author

Mr Paul Brennan, Translational Neurosurgery, Centre for Clinical Brain Sciences, Chancellor's Building, 49 Little France Crescent, Edinburgh, EH16 4SB, United Kingdom
Paul.brennan@ed.ac.uk
Tel: +44 (0) 131 537 1000

Word Count: 3651

Abstract Word Count: 300

For peer review only - http://bmjopen.bmj.com/site/about/guidelines.xhtml

ABSTRACT

Introduction

Fractures of the odontoid process frequently result from low impact falls in frail or older adults. These are increasing in incidence and importance as the population ages. In the UK, odontoid fractures in older adults are usually managed in hard collars to immobilise the fracture and promote bony healing. However, bony healing does not always occur in older adults, and bony healing is not associated with quality of life, functional, or pain outcomes. Further, hard collars can cause complications such as skin pressure ulcers, swallowing difficulties, and difficulties with personal care. We hypothesise that management with no immobilisation may be superior to management in a hard collar for older or frail adults with odontoid fractures.

Methods & Analyses

This is the protocol for the Duration of External Neck Stabilisation (DENS) trial - a non-blinded randomised controlled trial comparing management in a hard collar with management without a collar for older (≥65 years) or frail (Rockwood clinical frailty scale ≥5) adults with a new odontoid fracture. 887 neurologically intact participants with any odontoid process fracture type will be randomised to continuing with a hard collar (standard care) or removal of the collar (intervention). The primary outcome is quality of life measured using the EQ-5D-5L at 12 weeks. Secondary outcomes include pain scores, neck disability index, health and social care use and costs, and mortality.

Ethics & Dissemination

Informed consent for participation will be sought from those able to provide it. We will also include those who lack capacity to ensure representativeness of frail and acutely unwell older adults. Results will be disseminated via scientific publication, lay summary, and visual abstract. The DENS trial received a favourable ethical opinion from the Scotland A Research Ethics Committee (21/SS/0036) and the Leeds West Research Ethics Committee (21/YH/0141).

Registration Details

Registered at ClinicalTrials.gov (NCT04895644).

For peer review only

For peer review only - http://bmjopen.bmj.com/site/about/guidelines.xhtml

Strengths & Limitations of this study

- The DENS trial is a multi-centre non-blinded randomised controlled trial comparing the use of hard collars with no immobilisation.
- The primary outcome measure (EQ-5D-5L) is a quality of life measure relevant to the older or frail population.
- Health economic analysis and embedded qualitative study methods assess the wider implications of collar use in the older or frail population.
- Inclusion of all odontoid process fracture types, frail patients, and those with cognitive impairment will ensure the results are applicable to these populations.

The DENS trial does not directly compare surgical fixation and no immobilisation due to the preference for non-surgical management for older or frail patients in the UK.

Key Words
Odontoid process, odontoid peg, C2, cervical fracture, axis, dens, older adults, elderly, frailty, low-impact falls, cervical immobilisation, hard collar

For peer review only

For peer review only - http://bmjopen.bmj.com/site/about/guidelines.xhtml

INTRODUCTION

Background and Rationale

Odontoid process (dens) fractures occur following low impact falls in frail and older people, and are increasing in incidence as the population ages.[1-3] The Trauma Audit and Research Network (TARN) database identified at least 1,700 odontoid fractures each year in England and Wales, 85% in people over 65 years. One-year mortality following an odontoid fracture is 20-50%, similar to or higher than following a hip fracture, likely reflecting the underlying frailty and health status of those at risk of low impact falls.[4-7]

In the UK, 85-90% of frail or older people with a new odontoid fracture are managed non-operatively, most with 6-12 weeks immobilisation in a hard collar.[8-10] This aims to promote bony fusion and prevent neurological deterioration or instability pain.[1 ,5 ,11] However, hard collars only restrict 40-50% of neck movements,[12 ,13] and bony fusion rates of 20-80% are variable across fracture types, management, age, and frailty.[1 ,5 ,11 ,14-18] Further, bony fusion may not be associated with pain, quality of life, mortality, or functional outcomes in older people,[8 ,19 ,20] and late neurological deterioration is very rare.[5 ,11 ,21] Hard collars can cause skin pressure ulcers and difficulties with swallowing, breathing, and personal care, all of which can affect quality of life (QoL).[10 ,22] Additional health and social care input is often required to assist patients with activities of daily living. If fibrous union, or lack of bony fusion is an acceptable outcome and hard collars can negatively impact QoL, then management of an odontoid fracture with a hard collar may be causing unnecessary harm and negatively impacting on QoL in a group for whom the short to medium term QoL may be more important than long term outcomes.

Spinal surgeons responding to a UK survey variably reported managing odontoid fractures without any immobilisation or removing hard collars in frail or older patients who were unable to tolerate a collar, had a short life expectancy, or suffered complications.[10] Ninety percent supported randomisation to management with or without a collar to determine optimum treatment.[10] Further, feedback from patients managed in collars supports exploring whether collars are necessary for odontoid process fractures.

The Duration of External Neck Stabilisation (DENS) trial will test the hypothesis that management of odontoid process fractures without immobilisation is associated with improved QoL compared to management in a hard collar for 12 weeks.

Aims and Objectives

The primary aim is to determine the difference in QoL measured using the EQ-5D-5L at 12 weeks between frail or older patients with odontoid process fractures managed with a hard collar (standard care) or without any immobilisation (intervention).

Secondary aims include assessing differences in neck pain, functional recovery, complications, radiological findings, mortality, and overall health-care use and costs, up to one year post injury.

A nested qualitative study will explore trial recruitment experiences from the perspectives of patients, carers, and healthcare professionals, and explore adherence to group allocation.

For peer review only - http://bmjopen.bmj.com/site/about/guidelines.xhtml

Findings from the qualitative study will inform recruitment and consent procedures and aid interpretation of trial results.

METHODS AND ANALYSIS

Study design

This manuscript has been prepared in accordance with the SPIRIT checklist. The DENS trial is a non-blinded randomised controlled trial with a nested qualitative study comparing early hard collar removal (intervention) with treatment in a hard collar for 12 weeks (standard care) in older or frail adults with a new odontoid process fracture. Figure 1 shows the trial flow chart. The study will recruit from November 2021 to December 2023.

Study Eligibility

Inclusion Criteria:

- Aged ≥65 years, or Rockwood clinical frailty scale (CFS) of ≥5 (at least mildly frail: help needed in high order instrumental activities of daily living)[23]
- Recent odontoid process fracture of any type[24] secondary to low impact trauma (any degree of fracture angulation, displacement or canal narrowing)
- History of recent trauma (within 3 weeks)
- Recruited within 3 weeks of injury
- Determined by consultant spinal surgeon as suitable for standard treatment in hard collar or treatment without a collar

Exclusion Criteria:

- Fracture sustained in a high-impact injury
- New neurological deficit attributable to the fracture
- Unable to tolerate a hard collar
- Additional (non-odontoid process) cervical spine fracture not suitable for management without a hard collar
- Underlying condition with risk of spinal instability (e.g. ankylosing spondylitis)
- Fracture suspected to be older than three weeks at the time of assessment
- Not expected to survive to hospital discharge

A consultant radiologist report of an odontoid process fracture on CT and confirmation of eligibility by a consultant spinal surgeon (orthopaedic or neurosurgical) is required. Patients with additional cervical spine fractures are eligible provided the consultant spinal surgeon determines the fracture(s) are suitable for management without a hard collar. There are no exclusions for other injuries (e.g. fractured femur). Co-enrolment in other studies is permitted where this does not affect the DENS trial randomisation allocation or outcome measure assessment, and where this is not expected to burden the participant.

Participant Recruitment

Potential participants will be identified by clinicians following presentation to Emergency Departments (EDs) or acute assessment units, through review of acute cervical spine CT reports, acute hospital attendance, or through referrals or admissions to spinal services in the UK. An anonymised screening log will be kept.

For peer review only - http://bmjopen.bmj.com/site/about/guidelines.xhtml

Any neck stabilisation can be applied as part of standard care prior to randomisation, including; trauma collar, blocks, spinal board, padded hard collar (e.g. Miami J, Aspen, Philadelphia) or soft collar. Standard care treatment, such as removal from spinal boards and placement of a padded hard collar will occur as usual, and will not be delayed by trial assessment and participation.

Eligibility assessment and randomisation will take place as soon as possible after injury (target within 48 hours) to maximise the impact of study intervention. However, falls in older and frail people are usually multifactorial,[25 ,26] and conditions leading to the fall or resulting from the fall may affect capacity and fitness to participate. Recruitment can occur up to 3 weeks after the injury to provide flexibility for those presenting late, facilitate participation in those who are acutely unwell, and ensure time for involvement of patient representatives where needed.

The nested qualitative study will include healthcare professionals involved in patient recruitment, study participants, carers of participants lacking capacity, and those who decline trial participation.

Randomisation and Interventions
Web-based randomisation will occur with a minimisation algorithm based on:

- Age at randomisation (<75 versus ≥75 years)
- Odontoid Fracture type (I-III)[24]
- Frailty (CFS ≥5 versus <5)[23]

The first participant will be allocated to one arm with a probability of 0.5 and subsequent participants will be allocated with a probability of 0.8 to the group which minimises differences of the above variables between the two trial arms. Treatment allocation will be disclosed to the local research team via the web interface following randomisation.

The standard care arm will involve 12 weeks of hard collar treatment, following local usual choice of hard collar and local usual arrangements for collar care. For those allocated to the intervention (collar removal) arm, the collar will be removed as soon as possible, but may be weaned over several days if required for optimising analgesia. Removal of a collar prior to 12 weeks in the hard collar arm or wearing of a collar in the no collar arm will be recorded as a failure of adherence with explanation. Soft collars will not be used in either arm.

Outcome Measures
The primary outcome measure is the EQ-5D-5L at 12 weeks post-randomisation.
Secondary outcome measures are:

- 2 & 6 weeks: EQ-5D-5L, neck disability index (NDI), neck numeric pain scale
- 12 weeks: NDI, neck numeric pain scale, adverse events (AEs), compliance with hard collar use, analgesia use, length of hospital stay, discharge destination, bony fusion / stability on imaging, loss of muscle bulk in upper limbs using grip strength and bio-impedence

For peer review only - http://bmjopen.bmj.com/site/about/guidelines.xhtml

- 6 & 12 months: EQ-5D-5L, NDI, neck numeric pain scale, injury-related complications, mortality, health and social care visits/use, hospital admission or outpatient visits, health economics analysis

Trial assessments

Assessments and timing of data collection are shown in Table 1. Baseline data on demographics, comorbidities, injury, radiological findings and inpatient management will be recorded. Patient diaries will be used to collect pain scores, analgesia and collar use. Standard local clinical follow up will occur. Research assessments will be carried out at 2, 6 and 12 weeks by the local research team and by a blinded member of the central research team at 6 and 12 months. Assessments will occur via telephone interviews, postal or online questionnaires, or during standard care appointments. A proxy can provide information on a patient's behalf when required. Up to three reminder telephone calls will be made to maximise completion.

Table 1: **Timing of Study Assessments.**

Assessments undertaken as standard care are shown with x. Study specific assessments are shown with *. Assessments may occur within the following time windows: 2 weeks (±1 week), 6 and 12 weeks (± 2 weeks) and 6 and 12 months (± 4 weeks). Fracture type refers to that of Anderson & D'Alonzo.[24] (CFS: Rockwood clinical frailty scale;[23] NDI: neck disability index; AEs: adverse events)

	**Timing from Randomisation**						
	Baseline	Discharge	2 weeks	6 weeks	12 weeks	6 months	12 months
**Assessment**							
Eligibility Assessment	*						
Consent	*						
**Demographics**							
Baseline data	*						
CFS	*						
**Imaging**							
Fracture Type	x						
Imaging Type	x				x		
**Outcomes**							
EQ-5D-5L			*	*	*	*	*
EQ-VAS			*	*	*	*	*
Neck numeric pain scale			*	*	*	*	*
NDI			*	*	*	*	*
AEs		*			*		
Injury-related complications						*	*
Collar use		*	*	*	*		
Grip strength/bioimpedance	*				*		
Service use			*	*	*	*	*
Analgesia use			*	*	*		
Mortality					*	*	*

At centres with facilities for routine assessment of grip strength and bio-impedence, all participants will be invited to take part in this arm of the study. Loss of muscle bulk in the

For peer review only - http://bmjopen.bmj.com/site/about/guidelines.xhtml

upper limbs will be assessed over 12 weeks using hand grip strength measured with a dynamometer.[27] Bio-impedence measurements will be made at the wrist and ankle. Muscle mass and skeletal muscle index as a marker of frailty will be calculated as per Janssen et al.[28]

Approximately 25 patients in the collar arm will use temperature sensors to assess compliance with the collar (Thermochron iButtons).[29] These will be the first participants willing to participate at centres where the temperature sensors can be missioned. Long term follow up of mortality and hospital admissions using anonymised data without participant contact will occur for 5 years.

No study specific follow up imaging is mandatory. Participants will follow local clinical protocols for imaging. The modality and findings of all imaging undertaken will be recorded and imaging collected at the study centre for analysis where patient consent for transfer is given.

Qualitative study
Qualitative researchers will interview healthcare professionals about their recruitment experiences, perceptions of why patients decline or consent to participation, and reasons for not approaching eligible patients. Patients and caregivers will be interviewed at two time points – following randomisation and 12 weeks later. Post-randomisation interviews will explore reasons for taking part or declining participation, views about trial recruitment approaches, hopes and expectations regarding trial participation, and any anticipated difficulties or concerns adhering to treatment allocation. Follow-up interviews will explore treatment adherence, benefits or burdens of the allocated treatment, and perceptions of the impact on QoL and recovery. All interviews will use topic guides to help ensure the discussion remains relevant to addressing the study aims, while offering participants flexibility to raise issues they consider important, including those unforeseen at the study outset.

Sample Size
The recruitment target is 887 participants from around 25 UK sites over 2 years. An unadjusted sample size was calculated based on 90% power and a 5% significance level, using a two-sided two-sample t-test, for an effect size of 1/6 (0.05 minimum clinically important difference / 0.3 standard deviation) on the EQ-5D-5L at 12 weeks. This results in a sample size of 1514, increasing to 1893 assuming 20% missing data. This sample size can be reduced by accounting for correlation between baseline covariates (age, CFS) and 12 week EQ-5D-5L. It is anticipated that this correlation will be around 0.5, allowing a 25% reduction in sample size. Higher correlation will allow a larger reduction.[30] Further, the post-randomisation measures of EQ-5D-5L at 2, 6 and 12 weeks, are assumed to have a serial correlation of 0.44 which allows a further 37.5% reduction in sample size.[30 ,31] Including these reductions leads to the final target sample size of 887, with 1:1 allocation.

To check these assumptions, a sample size re-estimation will be modelled using a simulation approach based on 12 week outcome data from the first 300 participants.[30] Due to the conservative assumptions made, particularly the assumed standard deviation, the probability of increasing the sample size is small, and there is a larger probability it can be

For peer review only - http://bmjopen.bmj.com/site/about/guidelines.xhtml

reduced.

Based on audits in two UK neurosurgical units, and assuming a 50% recruitment rate with staggered site opening, the target recruitment rate is 1.6 patients per site per month. An internal pilot will assess recruitment feasibility, targeting 132 patients (15% total) across 18 centres in 9 months. Progression criteria for the pilot are shown in Table 2.

Table 2: Study Progression Criteria
Study progression criteria for the internal pilot to assess recruitment feasibility after the first 9 months of recruitment. The traffic light categories will lead to the following actions. Green: continue unchanged. Amber: make changes, including adding more sites or increasing the time period for recruitment. Red: continue stopping as study may not feasible, unless identifiable and rectifiable causes are identified. Adherence failure includes study withdrawals and cross-over (removing a collar in the collar arm, or wearing a collar in the no collar arm).

	Category		
	Red	Amber	Green
Total number recruited	≤108	109-131	≥132
Recruitment threshold (%)	≤82%	83%-99%	100%
Recruitment rate (patients per site per month)	1.07	1.33	1.63
Number of sites open	<5	5-10	>10
Adherence failure	>20%	11-20%	≤10%

The qualitative study will recruit approximately 20 healthcare professionals and 30 patients or carers from a cross section of sites. Purposive sampling will be used to ensure representation of different ages, socio-economic backgrounds, cognitions, and residential setting amongst patients. Sampling decisions may be revised in light of emerging findings.

Data Analysis Plan
The primary data analysis will be by intention to treat. A 5% two-sided significance level will be used throughout. A detailed statistical analysis plan will be developed by the study statisticians and finalised prior to locking of the trial database.

Analysis of the primary outcome will be a repeated measures analysis of covariance, including terms for treatment arm and the EQ-5D-5L responses at 2, 6 and 12 weeks. Adjustments will be made for the variables included in the randomisation minimisation algorithm. Adjustment for study site will be included as a random effect, if appropriate. The repeated measures approach enables estimation of an intervention effect at week 12 post-randomisation (primary outcome), and an overall assessment of the effect of the intervention over the 12-week period.

For peer review only - http://bmjopen.bmj.com/site/about/guidelines.xhtml

The 12-week NDI and VAS will be analysed in a similar manner to the EQ-5D-5L. It is anticipated that results will underpin any between group differences seen in EQ-5D-5L, confirming and strengthening the clinical interpretation of the findings. All other secondary outcomes will be analysed with statistical models appropriate to the distribution of the outcome, see Table 3 Where there are data recorded on multiple occasions post randomisation we may use an appropriate repeated measures model. Exploratory subgroup analyses by fracture type (I, II and III),[24] CFS (<5 versus ≥5)[23] and age (<75 versus ≥ 75 years) will be performed. The influence of any missing data on the robustness of the findings will be examined e.g. using multiple imputation models under a missing at random assumption. Screening logs will be analysed to assess generalisability of results.

Table 3: Possible Methods of Analysis

Methods to be used for analysing variables collected in the DENS study. A detailed statistical analysis plan will be published. (CFS = clinical frailty scale, NDI = neck disability index, NPRS = Numeric Pain Rating Scale.

Variable	Hypothesis	Outcome Measures	Analysis Methods
**Baseline data**	No difference between groups	Gender, Age, Comorbidities, Injury data, Radiological findings, Inpatient management, CFS	Categorical variables: absolute numbers and percentages. Continuous variables: mean, median, standard deviation (SD), interquartile range (IQR).
**Primary outcome**			
Health-related quality of life at 12 weeks	Clinically important difference between two groups with early collar removal superior	EQ-5D-5L (continuous)	Repeated measures analysis of covariance (ANCOVA) at 2, 6 and 12 weeks, adjusting for minimisation variables
**Secondary outcomes**			
Pain	No difference between groups	NPRS (ordinal)	Ordinal regression
Function	No difference between groups	EQ-VAS (continuous), NDI (continuous)	Repeated measures analysis of covariance at 2, 6 and 12 weeks,

For peer review only - http://bmjopen.bmj.com/site/about/guidelines.xhtml

			adjusting for minimisation variables
		Grip strength (continuous)	Student t test
		Bio impedance (ordinal)	Nonparametric methods; Mann-Whitney, Wilcoxon signed ranks test
Mortality	No difference between groups	All-cause mortality (binary)	Logistic regression, Chi-square
Collar use	Significantly greater in the collar group	Number of days of collar use (continuous)	Poisson regression
Health economics	Baysian assessment of likelihood incremental cost per QALY of either arm is below NICE Thresholds (£20k to £30k per QALY)	Service use & cost, QALYs (adjusted from EQ-5D-5L)<br><br>Incremental Cost Per QALY	Generalised linear lodelling of cost and QALYs.<br><br>Probabilistic sensitivity analysis via recycled predictions
**Sub-group analysis;**			
Fracture type (I, II and II)<br>CFS (<5 versus ≥5)<br><br>Age (<75 versus ≥ 75 years)	No difference<br><br>Better treatment effect in CFS ≥5<br>Better treatment effect in age ≥ 75		For each analysis, the subgroup variable and interaction term between intervention and subgroup variable will be included in the model.

Interview data will be analysed thematically using the method of constant comparison. To ensure rigour, several individuals will be involved in data analysis and coding. An interactive working group will be set up, comprising the qualitative research team, co-investigators, the trial manager, PPI representatives, and at least one healthcare professional from each of the sites involved in the pilot phase. A 'what, so what, now what' approach[32] will be used to translate qualitative findings and other experiences shared by group members into tangible recommendations.

A 12 month within trial health economic analysis will be undertaken based on National Institute for Health and Care Excellence (NICE) reference case recommendations to maximise UK policy relevance.[33] This will include: adoption of a National Health Service

For peer review only - http://bmjopen.bmj.com/site/about/guidelines.xhtml

(NHS) and Personal Social Services decision perspective; cost-utility approach for primary analysis (results presented in terms of incremental cost per quality adjusted life year (QALY), derived from EQ-5D-5L data with an area under the curve approach, omitting baseline); discount rate of 3.5% for both costs and QALYs (where applicable); and use of probabilistic sensitivity analysis, to generate cost effectiveness acceptability curves via the recycled predictions technique.[33 ,34] Choice of primary analysis cost per QALY threshold and EQ-5D-5L scoring algorithm will match NICE preferences at the time of data lock. Resource use will be combined with standard UK price weights to generate costs.[35 ,36] The latest financial year for which at least one study participant provides data and prices are available will be selected as base year. Univariate mean QALYs, resource use, and costs will be presented for each trial arm alongside differences in means and associated 95% confidence intervals. Multivariate analysis of both QALYs and total costs, will also be presented controlling for baseline costs where available, and minimisation variables. Missing data will be imputed using appropriate techniques depending on degree of missingness, likely multiple imputation by chained equations.

Patient and Public Involvement (PPI)
Patients and the public have played an active role in developing the grant application and project protocol, are co-applicants on the grant and are represented on the Trial Steering Committee. We asked older people with odontoid fractures and members of a PPI group for their opinion on EQ-5D-5L and NDI as possible primary outcome measures. Older patients with odontoid fractures managed to complete the EQ-5D-5L, whilst some struggled with the NDI, and described some of the NDI domains as irrelevant. PPI input identified frailty rather than age as more influential of daily function, leading to inclusion criteria including both. A lay group also reviewed and rewrote the lay summary of the trial and had input into its likely acceptability. At the end of the study, we will produce a plain language summary of the study results and a visual abstract, in conjunction with participants and our PPI partners.

ETHICS AND DISSEMINATION

The DENS trial received a favourable ethical opinion from the Scotland A Research Ethics Committee (REC) on the 10th June 2021 (21/SS/0036) and the Leeds West REC (on behalf of England and Wales) on the 29th July 2021 (21/YH/0141).

Participant Capacity and Consent
The study member obtaining consent will explain and clarify all relevant information, either face-to-face, or via telephone or video call, and give patients the time they need to decide whether to participate. Informed consent will be signed and dated by participants or their representative, and a study team member. Verbal consent from a participant or their representative, signed and witnessed by a study team member will also be valid. Participants may withdraw consent at any time without reason, without affecting their care.

Patients diagnosed with odontoid fractures may lack capacity. Recruitment of these patients is necessary to aid generalisability of the trial outcome and to allow equal access to any benefits of participation. All consent procedures will adhere to the Adults with Incapacity (Scotland) Act 2000, the 2005 Mental Capacity Act, the Mental Capacity Act (Northern

For peer review only - http://bmjopen.bmj.com/site/about/guidelines.xhtml

Ireland) 2016, and their codes of practice. For patients in Scotland who lack capacity, the patient's nearest relative, welfare attorney or guardian can provide consent. For patients in England, Wales and Northern Ireland who lack capacity, a personal consultee (relative or close friend) can provide consent. Where possible this will be written and signed, otherwise clear documentation of the discussion and named persons involved will be accepted.

If a participant without capacity regains capacity, informed consent will be sought to continue in the trial. In Scotland, if a participant has given informed consent, but loses capacity, they will remain in the study. In England, Wales, and Northern Ireland, if a participant has already given informed consent, but loses capacity, they will remain in the study only if consent is then given by a consultee.

Safety Considerations
Adverse Events (AEs) will be recorded up to 12-weeks. Expected AEs relating to hard collar use or odontoid process fractures include skin pressure ulcers, dysphagia, and recurrent falls. All AEs (expected or not) will be reviewed by the local principal investigator and reported to the data monitoring committee (DMC). Serious Adverse Events (SAEs) will also be reported to the trial sponsor, who will notify the REC. Outcome data, AEs and SAEs, will be routinely reviewed by the DMC. The DMC is independent and oversees trial safety and data monitoring. If the DMC assesses that the risk-benefit balance is significantly changed by any new safety information, amendments to the trial will be made.

The trial funder (NIHR) and co-sponsors (University of Edinburgh and NHS Lothian) did not influence trial design. The sponsor ensures that data collection, management and trial monitoring are conducted appropriately and has overall responsibility for the study. Neither the funder nor the sponsor will have ultimate authority over writing of the report or the decision to submit the report for publication.

The trial will be coordinated by the central trial team based at the Edinburgh Clinical Trials Unit (ECTU), overseen by a Trial Management Group consisting of key trial members. Regular reports will be made to the Trial Steering Committee.

The Protocol has been designed by the Chief Investigator and collaborators. The University of Edinburgh has insurance for negligent harm caused by poor protocol design, and participating sites have their own insurance and NHS indemnity for clinical negligence and other negligent harm.

Data Management

Participant records will be identified using a unique study identification number. All records will be kept in a secure storage area with limited access. Data will be entered anonymously into a secure database created and maintained by ECTU, stored on a secure server at The University of Edinburgh compliant with all relevant regulations. Trained and delegated members of the research team will have password protected logins for their own centre. Data generated from the iButton sensors is anonymised using a numerical code, which will be recorded on the database. Interviews for the qualitative study will be digitally recorded using a device encrypted to AES256 standard. Imaging will be stored and transferred within the NHS network. Only anonymised scans will be processed outside the NHS network. All

For peer review only - http://bmjopen.bmj.com/site/about/guidelines.xhtml

investigators and study site staff will comply with the requirements of the appropriate data protection legislation (including the General Data Protection Regulation and Data Protection Act 2018) regarding collection, storage, processing and disclosure of personal information. Published results will not contain any personal data and individuals will not be identifiable. All trial related data will be archived for five years.

Study Dissemination

Ownership of the data arising from this study resides with the study team. The primary trial publication will be drafted by a writing committee and will recognise the work of those involved in the trial. PIs and investigators on delegation logs for patient recruitment will be invited as collaborators on the primary trial publication. Trial results will be published in a peer-reviewed journal, presented at meetings and published as a plain language summary and visual abstract.

Data Availability Statement

Anonymised patient-level data will be available on reasonable request to the Chief Investigator (paul.brennan@ed.ac.uk). The trial protocol and statistical analysis plans will be published and available prior to trial completion.

Study Registration

Registered at ClinicalTrials.gov (NCT04895644)

AUTHORS' CONTRIBUTIONS

JW, EE, PCC, PFXS, PMB contributed to the conception of the study. JW, EE, PB, JB, PCC, GC, HE, CK, SK, JL, DL, JN, AN, MJR, SDS, PFXS, AS, JT, and PBM all designed the protocol. JW, EE, JB, PCC, HE, CK, JL, DJL, MJR, SDS, and AS drafted the protocol. JW, EE, PB, JB, PCC, GC, HE, CK, SK, JL, DJL, JN, AN, MJR, SDS, PFXS, AS, JT, and PMB revised the manuscript critically for intellectual content and approved the final version for publication. PMB agrees to be accountable for all aspects of the work in ensuring that questions related to the accuracy or integrity of any part of the work are appropriately investigated and resolved.

ACKNOWLEDGEMENTS

We thank all patients, lay members, and members of patient and public involvement groups who gave feedback to improve the study and design a study that is relevant and helpful for older or frail patients with odontoid process fractures.

FUNDING STATEMENT

This work was supported by the National Institute for Health Research (NIHR) Health Technology Assessment (HTA) grant number 131118. EE, JW, MJR, CK, JN, DJL, SDS, and PFXS are supported by this grant. The funder had no role in the study design, writing of the protocol, or the decision to submit the paper for publication.

For peer review only - http://bmjopen.bmj.com/site/about/guidelines.xhtml

COMPETING INTERESTS STATEMENT
MJR is supported by an NHS Research Scotland Career Researcher Clinician award. EE is supported by Plymouth Charitable Fund and a University of Plymouth pump priming grant. All other authors declared no competing interests.

FIGURE LEGENDS

Figure 1: DENS Trial Flowchart

NDI: neck disability index; VAS: visual analogue scale; CT: computed tomography; C-spine: cervical spine; DISH: diffuse idiopathic skeletal hyperostosis

For peer review only

For peer review only - http://bmjopen.bmj.com/site/about/guidelines.xhtml

REFERENCES

1. Iyer S, Hurlbert RJ, Albert TJ. Management of Odontoid Fractures in the Elderly: A Review of the Literature and an Evidence-Based Treatment Algorithm. *Neurosurgery* 2018;82(4):419-30. doi: 10.1093/neuros/nyx546 [published Online First: 2017/11/23]
2. Kehoe A, Smith JE, Edwards A, et al. The changing face of major trauma in the UK. *Emerg Med J* 2015;32(12):911-5. doi: 10.1136/emermed-2015-205265 [published Online First: 2015/11/26]
3. Smith HE, Kerr SM, Fehlings MG, et al. Trends in epidemiology and management of type II odontoid fractures: 20-year experience at a model system spine injury tertiary referral center. *J Spinal Disord Tech* 2010;23(8):501-5. doi: 10.1097/BSD.0b013e3181cc43c7 [published Online First: 2010/10/14]
4. Cooper Z, Mitchell SL, Lipsitz S, et al. Mortality and Readmission After Cervical Fracture from a Fall in Older Adults: Comparison with Hip Fracture Using National Medicare Data. *J Am Geriatr Soc* 2015;63(10):2036-42. doi: 10.1111/jgs.13670 [published Online First: 2015/10/13]
5. Graffeo CS, Perry A, Puffer RC, et al. Deadly falls: operative versus nonoperative management of Type II odontoid process fracture in octogenarians. *Journal of neurosurgery Spine* 2017;26(1):4-9. doi: 10.3171/2016.3.spine151202 [published Online First: 2016/08/20]
6. Morri M, Ambrosi E, Chiari P, et al. One-year mortality after hip fracture surgery and prognostic factors: a prospective cohort study. *Sci Rep* 2019;9(1):18718. doi: 10.1038/s41598-019-55196-6 [published Online First: 2019/12/12]
7. Sheikh HQ, Athanassacopoulos M, Doshi AB, et al. Early mortality and morbidity following a type II odontoid fracture in the elderly. *The surgeon : journal of the Royal Colleges of Surgeons of Edinburgh and Ireland* 2018;16(5):297-301. doi: 10.1016/j.surge.2018.03.007 [published Online First: 2018/04/17]
8. McIlroy S, Lam J, Khan MF, et al. Conservative Management of Type II Odontoid Fractures in Older People: A Retrospective Observational Comparison of Osseous Union Versus Nonunion. *Neurosurgery* 2020 doi: 10.1093/neuros/nyaa256 [published Online First: 2020/06/23]
9. Watts A, Athanassacopoulos M, Breakwell L, et al. Management of C2 Odontoid peg fragility fractures - A UK survey of spinal surgeons. British Association of Spine Surgeons Conference. Brighton, UK, 2019.
10. Woodfield J. Odontoid Fracture Survey Results (Personal communication), 2019.
11. Pal D, Sell P, Grevitt M. Type II odontoid fractures in the elderly: an evidence-based narrative review of management. *European spine journal : official publication of the European Spine Society, the European Spinal Deformity Society, and the European Section of the Cervical Spine Research Society* 2011;20(2):195-204. doi: 10.1007/s00586-010-1507-6 [published Online First: 2010/09/14]
12. Lunsford TR, Davidson M, Lunsford BR. The effectiveness of four contemporary orthoses in restricting cervical motion. *Journal of Prosthetics and Orthotics* 1994;6(4):93-99.
13. Whitcroft KL, Massouh L, Amirfeyz R, et al. A comparison of neck movement in the soft cervical collar and rigid cervical brace in healthy subjects. *Journal of manipulative and physiological therapeutics* 2011;34(2):119-22. doi: 10.1016/j.jmpt.2010.12.007 [published Online First: 2011/02/22]

For peer review only - http://bmjopen.bmj.com/site/about/guidelines.xhtml

14. De Bonis P, Iaccarino C, Musio A, et al. Functional Outcome of Elderly Patients Treated for Odontoid Fracture: A Multicenter Study. *Spine* 2019;44(13):951-58. doi: 10.1097/brs.0000000000002982 [published Online First: 2019/06/18]
15. Fehlings MG, Arun R, Vaccaro AR, et al. Predictors of treatment outcomes in geriatric patients with odontoid fractures: AOSpine North America multi-centre prospective GOF study. *Spine* 2013;38(11):881-6. doi: 10.1097/BRS.0b013e31828314ee [published Online First: 2013/03/06]
16. Graffeo CS, Perry A, Puffer RC, et al. Odontoid Fractures and the Silver Tsunami: Evidence and Practice in the Very Elderly. *Neurosurgery* 2016;63 Suppl 1:113-17. doi: 10.1227/neu.0000000000001279 [published Online First: 2016/07/12]
17. Harrop JS, Hart R, Anderson PA. Optimal treatment for odontoid fractures in the elderly. *Spine* 2010;35(21 Suppl):S219-27. doi: 10.1097/BRS.0b013e3181f32716 [published Online First: 2010/10/15]
18. Smith HE, Kerr SM, Maltenfort M, et al. Early complications of surgical versus conservative treatment of isolated type II odontoid fractures in octogenarians: a retrospective cohort study. *J Spinal Disord Tech* 2008;21(8):535-9. doi: 10.1097/BSD.0b013e318163570b [published Online First: 2008/12/06]
19. Lofrese G, Musio A, De Iure F, et al. Type II odontoid fracture in elderly patients treated conservatively: is fracture healing the goal? *European spine journal : official publication of the European Spine Society, the European Spinal Deformity Society, and the European Section of the Cervical Spine Research Society* 2019;28(5):1064-71. doi: 10.1007/s00586-019-05898-2 [published Online First: 2019/01/24]
20. Perry A, Graffeo CS, Carlstrom LP, et al. Fusion, Failure, Fatality: Long-term Outcomes After Surgical Versus Nonoperative Management of Type II Odontoid Fracture in Octogenarians. *World neurosurgery* 2018;110:e484-e89. doi: 10.1016/j.wneu.2017.11.020 [published Online First: 2017/11/21]
21. Crockard HA, Heilman AE, Stevens JM. Progressive myelopathy secondary to odontoid fractures: clinical, radiological, and surgical features. *J Neurosurg* 1993;78(4):579-86. doi: 10.3171/jns.1993.78.4.0579 [published Online First: 1993/04/01]
22. Peck GE, Shipway DJH, Tsang K, et al. Cervical spine immobilisation in the elderly: a literature review. *British journal of neurosurgery* 2018;32(3):286-90. doi: 10.1080/02688697.2018.1445828 [published Online First: 2018/03/01]
23. Rockwood K, Song X, MacKnight C, et al. A global clinical measure of fitness and frailty in elderly people. *CMAJ* 2005;173(5):489-95. doi: 10.1503/cmaj.050051
24. Anderson LD, D'Alonzo RT. Fractures of the odontoid process of the axis. *The Journal of bone and joint surgery American volume* 1974;56(8):1663-74. [published Online First: 1974/12/01]
25. Hopewell S, Adedire O, Copsey BJ, et al. Multifactorial and multiple component interventions for preventing falls in older people living in the community. *The Cochrane database of systematic reviews* 2018;7:Cd012221. doi: 10.1002/14651858.CD012221.pub2 [published Online First: 2018/07/24]
26. Rubenstein LZ. Falls in older people: epidemiology, risk factors and strategies for prevention. *Age Ageing* 2006;35 Suppl 2:ii37-ii41. doi: 10.1093/ageing/afl084 [published Online First: 2006/08/24]
27. Bohannon RW. Hand-grip dynamometry predicts future outcomes in aging adults. *J Geriatr Phys Ther* 2008;31(1):3-10. doi: 10.1519/00139143-200831010-00002

For peer review only - http://bmjopen.bmj.com/site/about/guidelines.xhtml

28. Janssen I, Heymsfield SB, Baumgartner RN, et al. Estimation of skeletal muscle mass by bioelectrical impedance analysis. *J Appl Physiol (1985)* 2000;89(2):465-71. doi: 10.1152/jappl.2000.89.2.465
29. Benish BM, Smith KJ, Schwartz MH. Validation of a miniature thermochron for monitoring thoracolumbosacral orthosis wear time. *Spine* 2012;37(4):309-15. doi: 10.1097/BRS.0b013e31821e1488 [published Online First: 2011/05/05]
30. Posch M, Klinglmueller F, König F, et al. Estimation after blinded sample size reassessment. *Stat Methods Med Res* 2018;27(6):1830-46. doi: 10.1177/0962280216670424 [published Online First: 2016/10/05]
31. Frison L, Pocock SJ. Repeated measures in clinical trials: analysis using mean summary statistics and its implications for design. *Stat Med* 1992;11(13):1685-704. doi: 10.1002/sim.4780111304 [published Online First: 1992/09/30]
32. Breckenridge JP, Gianfrancesco C, de Zoysa N, et al. Mobilising knowledge between practitioners and researchers to iteratively refine a complex intervention (DAFNEplus) pre-trial: protocol for a structured, collaborative working group process. *Pilot Feasibility Stud* 2018;4:120. doi: 10.1186/s40814-018-0314-4 [published Online First: 2018/07/11]
33. NICE. Guide to the methods of technology appraisal. http://wwwniceorguk/article/pmg9/resources/non-guidance-guide-to-the-methods-of-technology-appraisal-2013-pdf, 2013.
34. Glick HA, Doshi JA, Sonnad AA, et al. Economic Evaluation in Clinical Trials. Oxford: Oxford University Press 2007.
35. Curtis L, Burns A. Unit Costs of Health and Social Care 2019. Canterbury: Personal Social Services Reseach Unit, 2018.
36. Department of Health. The Reference Costs Publications, 2019.

For peer review only - http://bmjopen.bmj.com/site/about/guidelines.xhtml

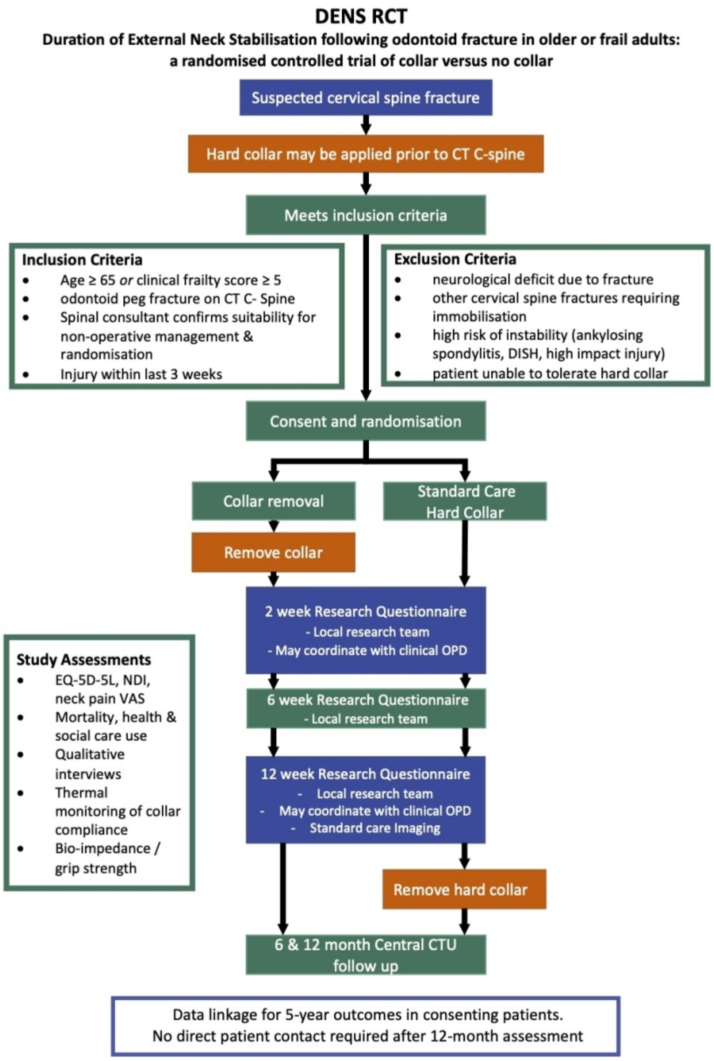

Figure 1: DENS Trial Flowchart

NDI: neck disability index; VAS: visual analogue scale; CT: computed tomography; C-spine: cervical spine; DISH: diffuse idiopathic skeletal hyperostosis

419x594mm (72 x 72 DPI)

SPIRIT

STANDARD PROTOCOL ITEMS: RECOMMENDATIONS FOR INTERVENTIONAL TRIALS

SPIRIT 2013 Checklist: Recommended items to address in a clinical trial protocol and related documents*

Section/item	Item No	Description	Page
**Administrative information**			
Title	1	Descriptive title identifying the study design, population, interventions, and, if applicable, trial acronym	1
Trial registration	2a	Trial identifier and registry name. If not yet registered, name of intended registry	2
	2b	All items from the World Health Organization Trial Registration Data Set	1-16
Protocol version	3	Date and version identifier	1
Funding	4	Sources and types of financial, material, and other support	12
Roles and responsibilities	5a	Names, affiliations, and roles of protocol contributors	1
	5b	Name and contact information for the trial sponsor	12
	5c	Role of study sponsor and funders, if any, in study design; collection, management, analysis, and interpretation of data; writing of the report; and the decision to submit the report for publication, including whether they will have ultimate authority over any of these activities	12
	5d	Composition, roles, and responsibilities of the coordinating centre, steering committee, endpoint adjudication committee, data management team, and other individuals or groups overseeing the trial, if applicable (see Item 21a for data monitoring committee)	11-12
**Introduction**			
Background and rationale	6a	Description of research question and justification for undertaking the trial, including summary of relevant studies (published and unpublished) examining benefits and harms for each intervention	4
	6b	Explanation for choice of comparators	4
Objectives	7	Specific objectives or hypotheses	4
Trial design	8	Description of trial design including type of trial (eg, parallel group, crossover, factorial, single group), allocation ratio, and framework (eg, superiority, equivalence, noninferiority, exploratory)	5-10

For peer review only

Methods: Participants, interventions, and outcomes

Study setting	9	Description of study settings (eg, community clinic, academic hospital) and list of countries where data will be collected. Reference to where list of study sites can be obtained	5-6
Eligibility criteria	10	Inclusion and exclusion criteria for participants. If applicable, eligibility criteria for study centres and individuals who will perform the interventions (eg, surgeons, psychotherapists)	5
Interventions	11a	Interventions for each group with sufficient detail to allow replication, including how and when they will be administered	6
	11b	Criteria for discontinuing or modifying allocated interventions for a given trial participant (eg, drug dose change in response to harms, participant request, or improving/worsening disease)	6
	11c	Strategies to improve adherence to intervention protocols, and any procedures for monitoring adherence (eg, drug tablet return, laboratory tests)	7
	11d	Relevant concomitant care and interventions that are permitted or prohibited during the trial	6-8
Outcomes	12	Primary, secondary, and other outcomes, including the specific measurement variable (eg, systolic blood pressure), analysis metric (eg, change from baseline, final value, time to event), method of aggregation (eg, median, proportion), and time point for each outcome. Explanation of the clinical relevance of chosen efficacy and harm outcomes is strongly recommended	6
Participant timeline	13	Time schedule of enrolment, interventions (including any run-ins and washouts), assessments, and visits for participants. A schematic diagram is highly recommended (see Figure)	Table 1 Figure 1
Sample size	14	Estimated number of participants needed to achieve study objectives and how it was determined, including clinical and statistical assumptions supporting any sample size calculations	8
Recruitment	15	Strategies for achieving adequate participant enrolment to reach target sample size	8

Methods: Assignment of interventions (for controlled trials)

Allocation:

Sequence generation	16a	Method of generating the allocation sequence (eg, computer-generated random numbers), and list of any factors for stratification. To reduce predictability of a random sequence, details of any planned restriction (eg, blocking) should be provided in a separate document that is unavailable to those who enrol participants or assign interventions	6

For peer review only - http://bmjopen.bmj.com/site/about/guidelines.xhtml

Allocation concealment mechanism	16b	Mechanism of implementing the allocation sequence (eg, central telephone; sequentially numbered, opaque, sealed envelopes), describing any steps to conceal the sequence until interventions are assigned	6
Implementation	16c	Who will generate the allocation sequence, who will enrol participants, and who will assign participants to interventions	6
Blinding (masking)	17a	Who will be blinded after assignment to interventions (eg, trial participants, care providers, outcome assessors, data analysts), and how	6
	17b	If blinded, circumstances under which unblinding is permissible, and procedure for revealing a participant's allocated intervention during the trial	6
**Methods: Data collection, management, and analysis**			
Data collection methods	18a	Plans for assessment and collection of outcome, baseline, and other trial data, including any related processes to promote data quality (eg, duplicate measurements, training of assessors) and a description of study instruments (eg, questionnaires, laboratory tests) along with their reliability and validity, if known. Reference to where data collection forms can be found, if not in the protocol	Table 1 Figure 1
	18b	Plans to promote participant retention and complete follow-up, including list of any outcome data to be collected for participants who discontinue or deviate from intervention protocols	6,7,11
Data management	19	Plans for data entry, coding, security, and storage, including any related processes to promote data quality (eg, double data entry; range checks for data values). Reference to where details of data management procedures can be found, if not in the protocol	11-12
Statistical methods	20a	Statistical methods for analysing primary and secondary outcomes. Reference to where other details of the statistical analysis plan can be found, if not in the protocol	9-10
	20b	Methods for any additional analyses (eg, subgroup and adjusted analyses)	9-10
	20c	Definition of analysis population relating to protocol non-adherence (eg, as randomised analysis), and any statistical methods to handle missing data (eg, multiple imputation)	9-10
**Methods: Monitoring**			
Data monitoring	21a	Composition of data monitoring committee (DMC); summary of its role and reporting structure; statement of whether it is independent from the sponsor and competing interests; and reference to where further details about its charter can be found, if not in the protocol. Alternatively, an explanation of why a DMC is not needed	11

For peer review only - http://bmjopen.bmj.com/site/about/guidelines.xhtml

	21b	Description of any interim analyses and stopping guidelines, including who will have access to these interim results and make the final decision to terminate the trial	7-8 Table 2
Harms	22	Plans for collecting, assessing, reporting, and managing solicited and spontaneously reported adverse events and other unintended effects of trial interventions or trial conduct	11
Auditing	23	Frequency and procedures for auditing trial conduct, if any, and whether the process will be independent from investigators and the sponsor	11
**Ethics and dissemination**			
Research ethics approval	24	Plans for seeking research ethics committee/institutional review board (REC/IRB) approval	10
Protocol amendments	25	Plans for communicating important protocol modifications (eg, changes to eligibility criteria, outcomes, analyses) to relevant parties (eg, investigators, REC/IRBs, trial participants, trial registries, journals, regulators)	12
Consent or assent	26a	Who will obtain informed consent or assent from potential trial participants or authorised surrogates, and how (see Item 32)	10-11
	26b	Additional consent provisions for collection and use of participant data and biological specimens in ancillary studies, if applicable	N/A
Confidentiality	27	How personal information about potential and enrolled participants will be collected, shared, and maintained in order to protect confidentiality before, during, and after the trial	11-12
Declaration of interests	28	Financial and other competing interests for principal investigators for the overall trial and each study site	13
Access to data	29	Statement of who will have access to the final trial dataset, and disclosure of contractual agreements that limit such access for investigators	11-12
Ancillary and post-trial care	30	Provisions, if any, for ancillary and post-trial care, and for compensation to those who suffer harm from trial participation	N/A
Dissemination policy	31a	Plans for investigators and sponsor to communicate trial results to participants, healthcare professionals, the public, and other relevant groups (eg, via publication, reporting in results databases, or other data sharing arrangements), including any publication restrictions	12
	31b	Authorship eligibility guidelines and any intended use of professional writers	12
	31c	Plans, if any, for granting public access to the full protocol, participant-level dataset, and statistical code	12

For peer review only

For peer review only - http://bmjopen.bmj.com/site/about/guidelines.xhtml

Appendices

Informed consent materials	32	Model consent form and other related documentation given to participants and authorised surrogates
Biological specimens	33	Plans for collection, laboratory evaluation, and storage of biological specimens for genetic or molecular analysis in the current trial and for future use in ancillary studies, if applicable

*It is strongly recommended that this checklist be read in conjunction with the SPIRIT 2013 Explanation & Elaboration for important clarification on the items. Amendments to the protocol should be tracked and dated. The SPIRIT checklist is copyrighted by the SPIRIT Group under the Creative Commons "Attribution-NonCommercial-NoDerivs 3.0 Unported" license.

For peer review only
